# Supplementary material for: ‘Children awaken by playing’: a qualitative exploration of caregivers’ norms, beliefs and practices related to young children’s learning and early childhood development in rural Burkina Faso
Source: BMJ Open. 2023 Oct 29;13(10):e075675. doi: 10.1136/bmjopen-2023-075675 (PMC10619012; doi:10.1136/bmjopen-2023-075675)
Supplement: Supplementary data [file bmjopen-2023-075675supp001.pdf]

Guide for In-depth interviews with Families on Care-giving

Welcome. We would like to understand more about how young children are cared for in your community to help us design a radio campaign that focuses on child development.

We would like to learn from you about current care giving practices, what you believe makes your child healthy and what your hopes are for your children.

[Reminder to interviewer: Questions should relate to the focus child less than 3 years of age]

Theme 1: Socio-demographic and interview information

1.1 ID:
1.2 Interview date:
1.3 Interview start time:
1.4 Interview end time:
1.5 Age:
1.6 Marital status:
1.7 Ethnicity/religion:
1.8 Last completed level of education:
1.9 Main occupation:
1.10 Total number of children:
1.11 Sex of baby:
1.12 Age of baby:
1.13 Interviewer code:
1.14 Tape recording number:

Theme 2: Typical day

1. Who has the main responsibility of caring for your child? What responsibilities do they have?

2. Who else is the child with during a typical day? How often and how long for? What do they do with the child?
   a. **Probe:** role of grandmother, father, siblings, neighbours

3. What are the key events in your child's day? [e.g. bathing, massage/ lotion, feeding].

4. Would you like to change the amount of time you spend with your child (more? Less?)
   a. **Probe:** *If more*: What are the reasons you spend less time than you would like with your child? What would you like to do if you had more time?

Theme 3: Learning and playing

1. From what age do you think your child begins to learn new things?
   a. When do they start to learn and understand about the world around them?
   b. When do they start to understand words?

2. I would like to know a little bit about how your child learns new things?
   a. How does your child learn new behaviours or skills?
   b. How does your child learn what is good or wrong (moral values)?
   c. Hoes does your child learn new words and sounds?
   d. At what age will they learn most? Learn the quickest?

3. What are you excited about seeing them learn or do in the next few years?

4. Tell me about the times you play with your child.
    a. When?
    b. What or how do you play?

5. What are your child's favourite things to play or to play with?

6. Does anyone else play with your child?
    a. Who?
    b. When?
    c. What or how do they play?

7. Does your child play on their own?
    a. When?
    b. What or how do they play?

8. What do you think about using household things such as bowls, bottles and spoons as play things?

9. Do you think your child learns anything while they play?
    a. If yes, what do you think your child learns when she/he plays?

10. Tell me about the times you talk to your child?
    a. What type of things do you talk to them about?
    b. Are they able to communicate back?

11. Do you tell your child stories/songs/rhymes?
    a. Why/why not?
    b. Can you share some of your favourite stories/songs/rhymes with me?

12. Do you look at books with your child?
    a. If you wanted to show your child some pictures of people, things or places what could you use?

Theme 4: Responsive caregiving

1. How do you make your child smile/laugh?

2. How do you show them that you love them?

3. How does your child let you know what they want or need?
    a. What do you do when they show you these things?

4. What sort of things make you unhappy with him/her?
    a. Can you share an example of the last time you were unhappy with your child?
    b. What did you do?

5. If your child does something good, how do you encourage them to do it again?

6. If your child does something that is unsafe, like get too close to the fire, how do you help them understand this is dangerous?

7. How is your child disciplined?
    a. Who disciplines them?
    b. Does everyone in the family agree about how to disciplined the child?

Theme 5: Aspirations and challenges

1. We all have hopes for our children's futures. What do you hope for your child as they grow older? What does a successful life look like for your child?

2. What can you do with your child to help them be that way? To meet those goals?

3. What kind of parent/caregiver do you want to be for your child?
    a. What problems to you face trying to be this type of parent?

4. Do you have any worries/concerns about your child's future?

5. Do you face any challenges when caring for your child? If yes, can you describe those challenges?

Theme 6: Additional notes for interviewer

1. Interviewer note here:
    a. Where interview took place
    b. Any interruptions
    c. Overall mood, openness, and engagement of participant

Draft Guide for focus group discussions with Families on Care-giving -FGD 1

Welcome. We would like to understand more about how young children are cared for in your community to help us design a radio campaign that focuses on child development.

We would like to discuss how children are cared for in this community

Theme 1: Socio-demographic and interview information

1.1 FGD ID:
1.2 FGD date (DD/MM/YYYY):
1.3 Village:
1.4 FGD start time (AM/PM):
1.5 FGD end time (AM/PM):
1.6 Interviewer code:
1.7 Note taker code
1.8 Tape recording number:

**Respondent number**	**Age**	**Marital Status**	**Education**	**Ethnicity/ religion**	**Number of children**	**Age of youngest child**

Theme 2: General child care

1. In general, who is responsible for caring for children the age of your child in this community? [Probes: Feeding, bathing, keeping them safe]

2. What role do fathers play in the care of children the age of your child? Do you think fathers would like to spend more or less time with their children?

3. What would the community say about a father who spent a lot of time with their young child? Would they see this as a good thing? A bad thing?

Theme 3: Playing

1. When do children start to learn?

2. What does it mean when a child is called smart? How does a child become smart?

3. At what age do children begin to play?

4. Is play important for a child? Why? [Probe: What do you think your child learns when she/he plays?]

5. What do you think would happen if people in this community started praising their child more for being ‘good’ and punished them less for being ‘bad’.

6. How would you describe a ‘difficult’ (troublesome) child? How does a ‘difficult’ child behave? What, generally, are the behaviors of a ‘difficult’ child? How would you say a child becomes ‘difficult’?

Theme 4: Radio clips

We would now like you to listen to three radio clips and we are interested to know what you think of these clips, whether you would want to do the activities described in them with your child or not. There is no right or wrong answer, we are only interested in your thoughts, so please be honest.

Follow up questions clip 1:

1. What do you think about the information shared in this clip?

2. Did you hear information that was new to you in this clip? What?

3. How does what you heard compare to what you currently do with your children?

Follow-up questions clips 2-4:
We just heard information about ***talking*** *to your child,* ***playing*** *with your child and* ***praising*** *your child.*

1. What do you think about the information shared in this clip?

2. Did you hear information that was new to you in this clip? What?

3. How does what you heard compare to what you currently do with your children?

4. Would anyone in your family or the community think any of these suggestions was bad or negative for your child if you did them? Why or why not?

We heard descriptions of three activities that you can do with your child to help them grow and develop. The three activities were:

1. *Talk to your child throughout the entire day. For example, you can talk to your baby during bathing, dressing, breastfeeding, diaper changes, chores (such as cooking, sweeping, and washing clothes), when sitting with the family.*
2. *Play with your child at least 15 minutes a day. Your child can play with things you have around the house - like bowls, spoons or cups - or toys you buy.*
3. *Encourage and congratulate your child. Encourage, applaud or congratulate your child when they do something that you like or learn a new skill. Hug and cuddle them a lot.*

We would like you to try these activities at least once a day. However, if you cannot do the activity every day – or at all – that is ok! This is not a test. We will meet in one week from today to discuss with you how the week went. We want to learn more about your experiences, even if you cannot do the activity every day or at all.

Next week we are going to meet here again and discuss how it went practising these suggestions with your baby/child.

Do you have any questions?

Draft Guide for focus group discussions with Families on Care-giving -FGD 2

Welcome. We are going to discuss you experiences trying the behaviours we heard about last week. The behaviours we asked you to try can be difficult to do during your busy week – or maybe your child did not like them. We want to hear about any of your experiences, even if you were not able to practice with your child.

Theme 1: Socio-demographic and interview information

a. FGD ID:
b. FGD date (DD/MM/YYYY):
c. Village:
d. FGD start time (AM/PM):
e. FGD end time (AM/PM):
f. Interviewer code:
g. Note taker code
h. Tape recording number:

**Respondent number**	**Age**	**Marital Status**	**Education**	**Ethnicity/ religion**	**Number of children**	**Age of youngest child**

1. We asked you to try some behaviours at home, how would you describe what you did differently?

2. Were there any behaviours you were not able to practise with your child or that were difficult to do?
   a. If yes, which behaviours?
   b. Why or why not? [probe to identify key challenges]

3. How did your child react to the behaviours?

4. Do you think doing these activities will help your child in any way? Why/why not? How?

5. How did you feel while talking with, playing with or praising your child?
   a. [**Probe**: Enjoyable? Fun? Neutral? Difficult? Annoying? Strange?]

6. How did your family members react?
   a. [**Probe**: other children? Spouse, co-wives, grandparents?].

7. Do you feel like you could incorporate the behaviours into your routine every day?
   a. If yes, when could you practise these behaviours with your child?
   b. Which ones would be easiest? Which was the hardest? Why?

8. Thinking about other families in your community, what is the main difficulty they will face in behaving in the ways we asked you to try?

9. Are there any behaviours they will not want to do?
