# Supplementary material for: ‘Children awaken by playing’: a qualitative exploration of caregivers’ norms, beliefs and practices related to young children’s learning and early childhood development in rural Burkina Faso
Source: BMJ Open. 2023 Oct 29;13(10):e075675. doi: 10.1136/bmjopen-2023-075675 (PMC10619012; doi:10.1136/bmjopen-2023-075675)
Supplement: Supplementary data [file bmjopen-2023-075675supp002.pdf]

Supplemental material

'Children awaken by playing': A qualitative exploration of caregivers' perceptions of young children's learning and early childhood development in rural Burkina Faso

Audio clips played for focus group discussion participants

→ The following text was audio recorded in French, Bissa and Dioula and played for participants in Focus Group Discussions. Participants were then asked for their reactions to the information shared in the recordings.

Clip 1: The basics and benefits of early childhood development
Children are curious from the start of their lives. They want to learn and understand the world. During the first five years of life, a child's brain grows faster than at any other time. So anything you say and do can help build a child's brain. Children's early experiences determine their brain development and give them the foundation for success in life. Talking and playing with the child from birth will make him active and intelligent.

Clip 2: Talk
Your children get it before they start talking. When they babble and twitter, they are looking to communicate or 'talk' in their own way with you. Talk to them often and imitate their sounds and expressions to see how they react - each of you can speak in turn!

Your baby loves the sound of your voice. Talk to your baby about everything you do during the day. Describe what you hear, see and smell around you. Point to objects and people and name them. This will facilitate their learning. You can talk to your baby during the bath, while you are dressing her, during meals, when you breastfeed her. You can also do this during household chores (such as cooking, cleaning, and laundry), or when you're sitting with your family and before bedtime. Tell your family to talk to the baby often, too. The more the baby is exposed to language (even before he can speak), the more words he will learn!

Clip 3: Play
It is through play that children learn about the world around them. Give your child your full attention for at least 15 minutes a day. Encourage him to look at your face or at the colored objects around you such as a picture, a plate or a mug.

When your baby gets older, help him discover the world around him by giving him items to hold in his hand to play. Look for safe objects or toys that he can hold in his hands and that he can shake or roll (such as a lime, orange, or any other round object in the house). You can also put beans in a small container with a lid that the baby can use and shake like a rattle. Have fun with him by hiding your face

behind your hands and making him laugh. This will help her understand that things don't go away even if they are hidden.

Take part in your child's play and exploration whenever you can. That way when he gets stuck or gets frustrated, you can help him kindly.

Clip 4: Encouragement and praise
Encouraging your child, at any age, shows him that you are happy with what he has done and it will make him want to do it again. Your child wants to know that you are happy with what he has done and the effort he has put in. Therefore, he will learn faster when you encourage him to try new things. It's always better to encourage your child to do the things you want them to do rather than constantly telling them not to do the things you don't want them to do! So encourage and praise him rather than saying negative words like “bad” or “don't do that.

“Instead, say “well done” and “well done!”, clap or smile when he does something that you like or when he is learning something new. Even babies who have not yet learned to speak are encouraged with positive words. So encourage and praise your baby when he tries to do new things (if he picks up objects or holds them in his hand, if he claps with his hands or tries to stand unaided). Use hugs, kisses and loving words with your child every day. Your child will then be brought in more often to do the activities you want him to do.
