# Supplementary material for: ‘Children awaken by playing’: a qualitative exploration of caregivers’ norms, beliefs and practices related to young children’s learning and early childhood development in rural Burkina Faso
Source: BMJ Open. 2023 Oct 29;13(10):e075675. doi: 10.1136/bmjopen-2023-075675 (PMC10619012; doi:10.1136/bmjopen-2023-075675)
Supplement: Supplementary data [file bmjopen-2023-075675supp003.pdf]

Table 1. Participant socio-demographics

Research Activity	Participant group	Total number of participants	Participant age range	Range of number of children**	Age ranges of children**			Child gender***	
					0-11 mo	*12-23 mo*	*24+ mo*	*Girl*	*Boy*
In-depth interviews	Mothers	16	20-40	1-7	6	8	2	9	7
	Fathers	16	23-59	1-15	8	7	1	6	10
*Focus Group Discussions**	Mothers	28	18-36	1-6	13	8	0		
	Fathers	26	24-58	1-10	17	9	0		
	Grandmothers	27	40-68	1-10	20	7	0		

* 5-7 participants per FGD
** For Grandmothers, refers to number/ ages of grandchildren
***Child gender only collected for IDIs
