# Supplementary material for: ‘Children awaken by playing’: a qualitative exploration of caregivers’ norms, beliefs and practices related to young children’s learning and early childhood development in rural Burkina Faso
Source: BMJ Open. 2023 Oct 29;13(10):e075675. doi: 10.1136/bmjopen-2023-075675 (PMC10619012; doi:10.1136/bmjopen-2023-075675)
Supplement: Author's manuscript [file bmjopen-2023-075675.draft_revisions.pdf]

BMJ Open

BMJ Open is committed to open peer review. As part of this commitment we make the peer review history of every article we publish publicly available.

When an article is published we post the peer reviewers' comments and the authors' responses online. We also post the versions of the paper that were used during peer review. These are the versions that the peer review comments apply to.

The versions of the paper that follow are the versions that were submitted during the peer review process. They are not the versions of record or the final published versions. They should not be cited or distributed as the published version of this manuscript.

BMJ Open is an open access journal and the full, final, typeset and author-corrected version of record of the manuscript is available on our site with no access controls, subscription charges or pay-per-view fees (http://bmjopen.bmj.com).

If you have any questions on BMJ Open's open peer review process please email info.bmjopen@bmj.com

BMJ Open

‘Children awaken by playing’: A qualitative exploration of caregivers’ perceptions of young children’s learning and early childhood development in rural Burkina Faso

Journal:	*BMJ Open*
Manuscript ID	bmjopen-2023-075675
Article Type:	Original research
Date Submitted by the Author:	15-May-2023
Complete List of Authors:	Dumbaugh, Mari; University College London, Institute for Global Health; Insight Impact Consulting<br>Belem, Mireille; Develpment Media International, Research<br>Kousse, Sylvain; Development Media International, Research<br>Ouoba, Patricia; Develpment Media International, Research<br>Sankoudouma, Adama ; Innovations for Poverty Action Francophone West Africa<br>Tchibozo, Achille; Innovations for Poverty Action Francophone West Africa<br>Fearon, Pasco; University of Cambridge, Department of Psychology; University College London, Centre for Family Research, Department of Psychology<br>Hollowell, Jennifer ; Development Media International Associates CIC,<br>Hill, Z; University College London Research Department of Epidemiology and Public Health, Institute for Global Health
Keywords:	Community child health < PAEDIATRICS, Paediatric neurology < PAEDIATRICS, Health Equity

SCHOLARONE™
Manuscripts

For peer review only - http://bmjopen.bmj.com/site/about/guidelines.xhtml

BMJ

I, the Submitting Author has the right to grant and does grant on behalf of all authors of the Work (as defined in the below author licence), an exclusive licence and/or a non-exclusive licence for contributions from authors who are: i) UK Crown employees; ii) where BMJ has agreed a CC-BY licence shall apply, and/or iii) in accordance with the terms applicable for US Federal Government officers or employees acting as part of their official duties; on a worldwide, perpetual, irrevocable, royalty-free basis to BMJ Publishing Group Ltd ("BMJ") its licensees and where the relevant Journal is co-owned by BMJ to the co-owners of the Journal, to publish the Work in this journal and any other BMJ products and to exploit all rights, as set out in our licence.

The Submitting Author accepts and understands that any supply made under these terms is made by BMJ to the Submitting Author unless you are acting as an employee on behalf of your employer or a postgraduate student of an affiliated institution which is paying any applicable article publishing charge ("APC") for Open Access articles. Where the Submitting Author wishes to make the Work available on an Open Access basis (and intends to pay the relevant APC), the terms of reuse of such Open Access shall be governed by a Creative Commons licence – details of these licences and which Creative Commons licence will apply to this Work are set out in our licence referred to above.

Other than as permitted in any relevant BMJ Author's Self Archiving Policies, I confirm this Work has not been accepted for publication elsewhere, is not being considered for publication elsewhere and does not duplicate material already published. I confirm all authors consent to publication of this Work and authorise the granting of this licence.

For peer review only

'*Children awaken by playing*': A qualitative exploration of caregivers' perceptions of young children's learning and early childhood development in rural Burkina Faso

Mari Dumbaugh[1,2], Mireille Belem[3], Sylvain Kousse[3], Patricia Ouoba[3], Adama Sankoudouma[4], Achille Mignondo Tchibozo[4], Pasco Fearon[5,6], Jennifer Hollowell[7], Zelee Hill[1] and the SUNRISE team

1. Institute for Global Health, University College London, UK
2. Insight Impact Consulting, Chicago, USA
3. Development Media International Burkina Faso
4. Innovations for Poverty Action, Francophone West Africa, Burkina Faso
5. Centre for Family Research, Department of Psychology, University of Cambridge, UK
6. Research Department of Clinical, Educational and Health Psychology, University College London, UK
7. Development Media International, London, UK

Corresponding author:
Dr Zelee Hill
z.hill@ucl.ac.uk

Institute for Global Health
University College London
30 Guilford Street
London, England
WC1N 1EH

For peer review only

‘*Children awaken by playing’:* A qualitative exploration of caregivers’ perceptions of young children’s learning and early childhood development in rural Burkina Faso

Keywords: Early childhood development; early learning; formative research; qualitative research; cultural adaptation; Burkina Faso; SUNRISE

Abstract

Introduction

Responsive caregiving and early learning activities positively impact developmental outcomes, with positive effects throughout the life-course. Supporting caregivers to provide this care is a key global strategy, but interventions should align with local values, beliefs and resources.

Methods

We conducted qualitative research in rural Burkina Faso to explore norms, beliefs, practices and aspirations around child development. We conducted 32 in-depth interviews with mothers and fathers of young children and 24 focus group discussions with mothers, fathers and grandmothers, which included trying behaviours and reporting on experiences. The research informed the development of SUNRISE, an early child development radio intervention.

Results

Caregivers described a process of “awakening”, through which children become aware of themselves and the world around them. When sufficiently awake children were able to learn through imitation, repetition, observation, explanation, correction, encouragement and play. Perceptions of the timing of awakening varied, but the ability to learn was thought to increase as children became older and more awake. Consequently, talking and playing with babies and younger children was perceived to have little developmental impact. Once sufficiently awake, children’s interactions with caregivers, alongside God-given intelligence, was believed to impact later behaviour and development. Despite these beliefs, caregivers felt their role in helping their children achieve later in life was to pay for education, save money, provide advice and be good role models – rather than through interaction and learning activities.

Caregivers who trialled interaction and learning activities reported positive experiences for themselves and their child, but interactions were often care-giver led and directive and play was often physical. A key barrier to carrying out the behaviours was a lack of time.

Conclusions

Exploring early childhood beliefs and practices can reveal important socio-cultural beliefs which if incorporated into program planning and implementation could help achieve more impactful, acceptable and equitable programs.

For peer review only - http://bmjopen.bmj.com/site/about/guidelines.xhtml

Strengths and limitations

- Purposive sampling and the use of multiple methods with a range of respondent groups and ethnicities allowed for triangulation and for varied views and experiences to be captured
- Rigour was enhanced by iterative reflexivity throughout and by double transcription and consensus coding of selected interviews
- We were unable to do any ethnographic work, which would have provided more depth, due to the security situation
- There is the potential for social desirability bias, especially when sharing experiences from the trials of improved practice, we ameliorated this through rapport building and by emphasising our desire to learn about all participant experiences, normalising challenges participants might experience and focused probing

Key messages

What is already known on this topic

Responsive caregiving and early learning activities positively impact early childhood development outcomes, as well as educational achievement and economic opportunities throughout the lifecourse. Globally, access and exposure to responsive care and learning activities early in life is inequitable, and approaches to child care can vary greatly between contexts. To adapt early childhood curricula from one context to another, it is best practice to combine existing beliefs and behaviours with evidence-based approaches.

What this study adds

This study expands the evidence base on normative early childhood practices and beliefs in a sub-Saharan African context. It demonstrates how reflexive formative qualitative research can be used to inform the development and adaptation of early childhood development curricula for diverse settings.

How this study might affect research, practice or policy

This study's design, analysis and practical application of results encourages researchers and practitioners to prioritise formative research on perceptions of learning and other aspects of early childhood development, and integrate findings into the development and implementation of early childhood curricula and programming, especially with marginalized populations and in non-Western contexts.

Introduction

Responsive caregiving and early learning activities positively impact early childhood development (ECD) outcomes, educational achievement and economic opportunities throughout the lifecourse [1–5]. Supporting caregivers to provide responsive care and learning activities during children's early years is considered a key strategy for achieving equitable health and development outcomes [1,5,6]. Effective delivery platforms include individualized home visits, group interventions and primary health care contacts [6]. The need to increase access to ECD opportunities in sub-Saharan Africa is well documented,

For peer review only - http://bmjopen.bmj.com/site/about/guidelines.xhtml

as only 47% of children aged three four years have adequate home stimulation and only 61% are developmentally on track – the lowest levels of any region [7].

Global guidelines for improving ECD require adaptations based on differences in socio-cultural contexts and ECD beliefs and practices [5,8–11]. Parental ethnotheories, or “the shared beliefs about the goals of child development and the socialisation practices that will achieve these goals” [12], can vary greatly between settings [3,12–18], and ECD interventions should account for and respect this diversity [18,19]. Best practice in developing and adaptating of ECD interventions is “blending…traditional childrearing practices and cultural beliefs with evidence-based approaches'' [8,16,17,20].

Formative research has informed the design of ECD interventions in low- and middle-income countries [9,11,16,16,21–27]. However, very few trials in sub-Saharan Africa reported using formative research or local community advisory boards in intervention design, and there has been little research exploring caregiver beliefs and perspectives on development and learning from birth [9,16,22,27–31]. We conducted formative qualitative research in rural Burkina Faso to explore ECD norms, beliefs and practices and aspirations of caregivers of children aged zero to three years. This research informed the development of *Scaling Up Nurturing Care, a Radio Intervention to Stimulate Early Childhood Development* (SUNRISE), running from 2022-2025 and offering caregivers evidence-based ECD information and strategies via radio broadcast to increase responsive engagement and early learning activities with young children (Clinicaltrials.gov identifier NCT05335395).

Methods

We conducted in-depth interviews (IDIs) with mothers and fathers and focus group discussions (FGDs) with mothers, fathers and grandmothers of children aged zero to three years in four villages in Balé and Boulgou provinces, Burkina Faso. Villages were safe for research teams and did not have exceptional characteristics, such as being located along a major thoroughfare or near a large health facility. We selected villages with a range of ethnicities to complement a previous data we had collected on the country’s dominant ethnic group (the Mossi) [22], allowing us to explore any differences between ethnic groups.

The Burkina Faso Institute for Health Science Research Ethics Committee and the University College London Research Ethics Committee granted ethical approval for this study.

Data collection

Local data collectors (women and men) had completed two to four years of undergraduate study. All had previous experience collecting qualitative data, and underwent three weeks of training for this study to review qualitative methods, ethics, research tools and transcription. We conducted a pilot study in peripheral neighbourhoods of Ouagadougou to test and finalise the semi-structured guides used to facilitate data collection.

Data were collected from March-April 2021. To recruit participants, fieldwork supervisors first introduced the study to village leaders. They were asked to identify a community member who was knowledgeable

For peer review only

about the community, but not a member of the village chief's family. The informant, often a community health worker, then accompanied the supervisor to relevant households where supervisors described their role as researchers, explained the study and provided an information sheet detailing the study objectives, confidentiality, participants' rights and research team contact numbers. Supervisors ensured potential participants were over 18 years of age and had a child zero to three years, and purposively selected participants for IDIs so that a range of children's ages were represented. For FGDs we recruited caregivers of children under 24 months as previous research shows caregivers are less likely to engage interactively with this age group in Burkina Faso [21,22]. No participants left the study once they agreed to participate.

Informed written consent was obtained from each participant. Research activities lasted 45-90 minutes, were conducted in Bissa or Dioula and audio recorded. Data collectors were paired with participants of their same gender. IDIs were conducted in a private place in participants' homes and explored typical daily routines, parents' perceptions of children's early learning and parents' short and long term aspirations for their children. Participants were given soap after each IDI to thank them for their time.

FGDs used an adapted *Trials of Improved Practice* (TIPS) methodology [22,32]. The first round of FGDs explored socio-cultural norms and beliefs around early child learning and caregiving. Then, participants listened to four informational recordings on the impact of early experiences on development, the importance of talking to children from birth, play as a vehicle for early learning and development and the benefits of frequent praise, encouragement and affection (see Supplementary Material). Participants shared their reactions to the spots, if they learned anything new and how community members might react to the suggestions. Finally, participants were invited to practise talking, playing or praising/ encouraging their child at home, and return for a second FGD in one week's time to share their experiences, challenges and child, family and community member reactions to the behaviours. One facilitator and one note taker held FGDs in classrooms or open air spaces away from other community members. Participants received money for transport.

Table 1. details participant socio-demographics. Participants were married or widowed and identified their religion as Muslim, Christian or animist/ traditional. Ethnic representation was diverse. Participants' education levels ranged from none to high school completion. Most educated participants had completed some or all of primary school. The vast majority were farmers.

Table 1. Participant socio-demographics

Research Activity	Participant group	Total number of participants	Age range	Range of number of children*	Age ranges of children*			Child gender**	
					0-11 mo	*12-23 mo*	*24+ mo*	*Girl*	*Boy*
In-depth	Mothers	16	20-40	1-7	6	8	2	9	7

For peer review only - http://bmjopen.bmj.com/site/about/guidelines.xhtml

For peer review only

interviews	Fathers	16	23-59	1-15	8	7	1	6	10
Focus Group Discussions	Mothers	28	18-36	1-6	13	8	0		
	Fathers	26	24-58	1-10	17	9	0		
	Grandmothers	27	40-68	1-10	20	7	0		

*For Grandmothers, refers to number/ ages of grandchildren
**Child gender only collected for IDIs

For peer review only

Data transcription and analysis

Audio data was translated and transcribed into French. To ensure data quality, the first interviews were double transcribed by data collectors in the field and trained, Ouagadougou-based transcriptionists. Senior researchers compared transcriptions for accuracy and gave translators feedback on the translation and each interviewer detailed feedback to enhance their reflexivity and improve probing. Throughout data collection a field-based researcher reviewed audio data and transcripts to ensure accurate translations.

Transcripts were analysed by francophone (MB, MD, SK, PO) and anglophone (ZH) senior researchers. We used a deductive-inductive approach to content analysis. First, using the semi-structured guides, a bilingual researcher developed separate code books for each data collection method. The code books were elaborated via double coding a selection of transcripts. Throughout analysis we held frequent meetings to discuss findings, clarify translations, iteratively refine the code books and determine when saturation was reached. Francophone researchers used NVIVO (released March 2020) to complete analysis; the Anglophone researcher coded translated transcripts (via Google Translate) by hand. Final transcriptions and findings were not sent to participants, however participants were given contact numbers of research team leads should they want to review data or results.

Participant and Public Involvement

Participants and/or the public were not involved in the design, or conduct, or reporting, or dissemination plans of this research.

Results

Our findings can be described by four major themes: perceptions of young children's consciousness; when and how children become conscious and learn; impacts of caregiver-child interactions; and, parents' aspirations for their children. We have noted where differences in results were observed by caregiver role. Caregiver perspectives reported in this paper did not differ by the caregiver's or child's gender (child's gender only collected for IDIs), or between ethnic groups.

Perceptions of young children's consciousness

Caregivers consistently conceptualised the development of child consciousness as a process of

For peer review only - http://bmjopen.bmj.com/site/about/guidelines.xhtml

"awakening". Awakening was used to describe a child becoming aware of themselves and the world around them: their intellectual development, increasing capacity to understand and act with intelligence, their ability to follow directions, to understand the difference between right and wrong and to act on socio-cultural norms such as greeting people, sharing food with others and bringing visitors water.

> *When a visitor arrives at the house, the child quickly goes to greet the visitor and gets water for him…The child tries to do what [adults] do around him. In doing so, he…[shows he] is truly awake.* (Grandmother; grandchild 4 months)

The process, timing and speed of awakening was, therefore, unique to each child and influenced by the "God-given" nature of the child, caregivers' behaviours, or a combination of both. These themes are expanded on throughout the following sections.

When and how children become conscious and learn

A major finding was caregivers' differing perceptions as to when children began to awaken and learn, varying from the earliest months of life to five years of age and the start of formal schooling. Caregivers said babies were not awake from birth as they were born without knowledge, memory or skills. Caregivers who said that babies were capable of learning new things in their first months cited various signs of early learning: smiling and laughing; "playing" with their hands; crying when they needed a diaper change; following people with their eyes; recognizing people; and acquiring new motor skills.

Perceptions of the timing of children's language comprehension also varied, from the earliest months of life to older than two years. Most caregivers thought that children usually start to comprehend language after about one year old, and show it by repeating and remembering words:

> *At 15 months the child begins to grasp some words and understand them. First he mixes up words, he calls people but if you are not paying attention you cannot understand who he is calling. At 16 or 17 months he begins to talk more clearly, he knows how to call his father and mother, and that progresses gradually until he is 20 or 24 months old.* (Father; child 33 months)

Fewer caregivers thought that preverbal children could learn to recognize and engage in forms of communication , such as understanding their mother's expressions and tones, recognizing when people were talking or being quiet and comprehending simple instructions from adults.

> *A child knows words even when he is small. Even at five months, if you say something, [the child] hears [what you are saying] but he cannot speak…Sometimes when you say certain things, like when you want to…hand him to someone…[the child] starts to cry. He cries because he understood something. [The child] begins…to hear words without you realising it.* (Father; child 11 months)

Caregivers said children learn to talk through interactions such as call and response, imitation, repetition and play with both adults and other children.

For peer review only - http://bmjopen.bmj.com/site/about/guidelines.xhtml

Talking to young babies in a conversant style was not common because of the perception that babies did not yet understand words and could not respond. Caregivers felt that talking to a preverbal baby was like talking to yourself. FGD participants highlighted this challenge after trying TIPS behaviours in their homes:

> *What was difficult for me was when the child said "ha ha ha!" That means that he wants to talk to us, but we do not understand what he says, so we tell him to stop his "ha ha's" and be quiet. Because as a Bissa saying goes, "A mute person cannot say to his grandmother, 'I want such and such a thing.'" [Talking with the child] was what was difficult.* (Grandmother; grandchild 1 month)

Caregivers also said some community members felt that talking to preverbal babies or playing with children was bizarre or a waste of time, with no real benefit to the young child.

> *I was sitting with my child in the middle of the day and I heard a knock at the door. The person [at the door] asked me who I was talking to, and I said I was talking to my baby....I told her my baby enjoys it when I talk to her … The person replied, "Ah, what does this little child know for you to speak [to her]?" I told her [my baby] understands and that's why I'm chatting with her. [My baby] laughs and she appreciates it, and I like it too. The person …[told] me that the [only] reason I am [talking to my baby] is that I don't have a job.* (Mother; child 1 month)

Despite divergence on when exactly awakening and language comprehension begin, a major finding was that caregivers agreed on the learning process itself. They said young children learn "little by little", learning more and more quickly as they awaken and are older. Children learnt through observation, recall, imitation and repetition of others; talk and explanations by caregivers; play on their own or with others; being corrected or encouraged; and at school through formal teaching. Talking to verbal children was believed to help children understand and learn words and recognize loved ones.

> *A child who is not used to having people talk to him will be less intelligent. But, a child who has always had people talking to him, that is like school for him and he will do remarkable things. You will see he is truly awakened.* (Grandmother; grandchild 1 month)

Play, or "children's work", alone or with peers was considered particularly important for development and learning as it helped awaken children and "open their spirit", helped children learn new words, expanded motor skills such as sitting up, clapping hands, crawling, walking and dancing and encouraged independence;; made children intelligent by providing new experiences, socialization with others, and taught children new ideas and lessons such as dangerous things to avoid (ie fire); and was an opportunity to practise life and work tasks through pretend games like cooking, playing shop, washing clothes and farming.

> *Play awakens children. If a child plays, he learns many things... Play also develops his intellectual capacities.* (Mother; child 2 months)

For peer review only

Children usually played independently or with siblings and peers. Play between adults and children was mostly physical such as bouncing up and down and other forms of play was not widespread but caregivers who tried playing with their children after FGDs had positive experiences.

Awakening and learning were at once influenceable and predestined. For example, while caregivers said play and talk could contribute to children's early or quick awakening, they also said these interactions would have limited influence until children were sufficiently awake (usually around two to three years old). Most caregivers also said "God given" or innate intelligence, character traits or circumstances determined children's development and learning.

> *God plots the future of each child.* (Grandmother; grandchild 4 months)

> *Some kids get better at a game the more they play. There are other children who sit and do not know how to play; everything is in the hands of God.* (Mother; child 21 months)

A child who "lacks blessings", for example, may be a difficult child or slow to follow instructions. Or, the quality of a woman's breast milk could positively or negatively impact her children's developmental achievements.

While recognizing that each child's developmental path was unique, caregivers had expectations around the timing of developmental milestones, and noticeable delays were cause for concern:

> *Children…do not all have the same growth rhythm. At six months, some are sitting up and crawling…There are other [children] who do not grow quickly. At one year they do not want to walk and it scares you…* (Mother; child 12 months)

Future impacts of caregiver-child interactions

Caregivers believed early experiences could impact a child's later behaviours and development, and that positive caregiver-child interactions were beneficial in the short and long terms.

> *Some women insult their children…, for example, 'Idiot, God will punish you!' But, the child hears what you say without being able to respond, and he grows up with the words spoken to him. So, if we speak to the child using [kind] words, he will grow up with that.* (Mother; child 18 months)

The perceived effects of disciplinary practices demonstrated the same. While caregivers universally agreed that sanctions and discipline were important aspects of child rearing, many also said that too frequent or too harsh discipline could have negative consequences for the child and their relationship with their parents well into adulthood.

The positive, long-term impacts of speaking to children softly, with positive words and encouragement were emphasised:

For peer review only - http://bmjopen.bmj.com/site/about/guidelines.xhtml

When you scold a child a lot, he gets confused; so you have to speak to him softly…this is when his intelligence will increase. (Mother; child under 6 months)

A mother has to congratulate the child when he does something good. This can bring the mother and child closer together. Whether the child is a girl or a boy, you have to encourage them. (Mother; child 7 months)

FGD participants said children reacted positively to talk, play and praise during the TIPS. Even very young babies reacted to ECD behaviours through direct eye contact, giggling, babbling and moving their arms and legs:

When he sees me he looks at me with big eyes and he moves [to show] he understands. When he sees me, he jumps, he laughs and he looks at me; so I know he understands. (Mother; child 5 months)

Older children's reactions to ECD behaviours tended to be more pronounced. After engaging in ECD behaviours, caregivers said older children responded more positively to them, were more aware of their presence, ran to greet them and asked them to play again. They said children were happier, more awake, had learnt to play or played better than before, increased their understanding and level of communication, had improved attitudes, followed directions, cried less and were more likely to interact and share with others.

Before [I implemented the ECD practices], …my child…did not want to share with anyone else. But, now, with the little time that I have played with him, he has gotten used to me…His mother bought him peanuts and…I asked him to give his grandmother one and he did. This is how I know he recognizes me now, as his father, thanks to the time I spent with him. (Father; child 30 months)

While these tangible changes were positive, the interactions caregivers described tended to be caregiver-led and directive in nature, rather than child-led and responsive to the child's signs and reactions. For example, talking at the child to give directions or monologue rather than engaging in a verbal or conversational exchange with the child, exclusively physical play (ie lifting child in air, tickling, blowing kisses) or giving children objects to keep them busy or console them rather than facilitating play with the child.

Parents' aspirations for their children

IDI participants were asked to describe their aspirations for their children as they grow up. In the short-term, parents wanted happy, healthy children who were successful at school. They wanted their children to be close to them, respectful of elders and visitors, helpful without being told what to do and good at following instructions and advice. They also described desirable character traits: independence, curiosity, politeness, active and alert (especially at school) and playful, as play signalled a healthy and happy child.

In the longer term, parents wished for their grown children to be healthy, continue practising their religion, marry a good person and have children themselves. Parents wanted children to be "better off"

For peer review only

than they were financially by acquiring money, land, a house, and animals, and hoped their children would be able to support them financially in old age and would visit them often. Many parents wanted their children to learn a trade, or to have a particular profession such as a government worker, health provider, entrepreneur, school teacher, religious teacher, soldier, or police.

> *If you put [your child] in school, he can be awakened and study, go farther than [you].* (Father; child 16 months)

Parents also hoped their children would become respected and forgiving community members, as their success as parents would be measured by their adult children's esteem in the community.

> *I would like her to be forgiving and that she would care for others. If you do not have a good heart and you do not care for others, no one will become close with you*. (Father; child 14 months)

Parents said they could help facilitate their children's success by paying for their education, saving money for them, guiding them with good, moral advice and being good role models, they rarely mention ECD behaviours as something they could do to facilitate success:.

> *It is up to us [parents] to show them, from a young age, what to do and what not to do to succeed in their life. All of this is through advice, if you do not guide your child, he won't succeed in life, and help you [in your old age].* (Mother; child 10 months)

Childrearing in extreme poverty presented parents with incessant challenges.

> *Raising children, it's not easy --- go to the hospital, eat, it's all very complicated for us. They say that [health services] are free, but they don't give you [free] medicines, so you are there suffering with your child. If you do not have money, it is exhausting to be a parent.* (Father; child 8 months)

A lack of food, treating household illnesses and injuries, having a baby before the youngest child had grown and maintaining patience with children also added to household difficulties.

Parents said their need to constantly seek out work, money and provide for the household left them with little time to practise ECD behaviours with their young children as often as they would like. Women in particular mentioned the effects of their high burden of housework, while some men said they often travelled for extended periods of time away from home.

> *My work is too much. During the day I have to prepare [food]; to pray; washing the children. Women [in our community] have a lot of work.* (Mother; child 12 months)

Discussion

Overall, caregivers felt that ECD behaviours would have a lasting, positive impact on their children's development in the short- and long-term. Caregivers described children awakening as becoming aware of themselves and the world around which them and consequently linked to understanding and the ability to learn. This concept of awakening echoes other descriptions of child development in sub-Saharan African settings which emphasise children's gradual acquisition of skills and knowledge as they develop

For peer review only - http://bmjopen.bmj.com/site/about/guidelines.xhtml

into “cultural agents” and community members [18], though this process of development was not necessarily linked to academic intelligence in all contexts [13]. Perceptions in our study of the timing and pace of children’s awakening, learning and of the impacts of caregiver behaviours, were said to be unique for each child and both predestined and influenceable. No differences in perceptions or beliefs were observed in our study by caregiver or child gender or between ethnic groups.

While caregivers in our study agreed that ECD behaviours had the potential to advance their children’s development, parenting in extreme poverty presented challenges to engaging consistently with children and time has been identified as a barrier to ECD behaviours in other African settings [27]. Some local beliefs could also hinder the practice of ECD behaviours. For example, the perceptions that the speed of awakening is in part predestined, that children cannot learn early in life and that play between adults and babies or young children is strange or caregivers who play with their children are wasting time. Caregivers tended to engage less with small babies, a finding which is supported by quantitative data from in Burkina Faso showing that few caregivers engaged in ECD-related behaviours with children aged zero to five months but that engagement increased with age [21] and qualitative data from Senegal where caregivers reported that it was inappropriate to talk to babies [16]. We found that play with caregivers was most often physical in nature and that interactions were caregiver-led.

This study expands the evidence base documenting ECD norms and beliefs for young children in sub-Saharan Africa, while also contributing to calls for culturally-aware and -relevant adaptations of ECD curricula and programming for non-Western contexts [13,14,17,33–35]. A number of authors argue that “imported” ECD curricula from Western socio-cultural frameworks “cannot fully meet the needs of African families” [36], as they will not reflect fundamental cultural and conceptual differences between those underlying Western parenting practises and the diverse parental ethnotheories across the Continent [17,18,34]. For these authors, implementing ECD curricula in African contexts which are informed solely by Western/ Eurocentric worldviews becomes, then, a question of ethics [14,17,18,35]. In practical terms, will caregivers practise behaviours which are incongruous with, even counter to, norms, beliefs, values or aspirations in their own lives?[33] While critical reflections of this sort are imperative, a robust empirical evidence base also demonstrates the positive effects of a standard set of ECD behaviours across a range of cultural contexts [1,4,37].

Our findings guided the adaptation of an ECD curriculum to the norms, values, beliefs and practises of a rural, sub-Saharan African context. The SUNRISE intervention design team, a mix of local and UK based mass media communication experts, used our findings to develop culturally-adapted ECD message briefs which highlight different ECD practices. The briefs were scripted into dramatic sketches, then locally produced and broadcast via radio in seven local languages multiple times a day, following the principles of the *Saturation+* approach to behaviour change [38]. Local radio presenters are also trained to host call-in talk shows on ECD-related topics [39]. The sketches and talk shows encourage responsive interactions and early learning activities with children from birth by integrating and validating local, normative perceptions and beliefs conducive to evidence-based ECD practices. For example, radio broadcasts were framed around the concept of “awakening” which was used to explain the principles of ECD and encourage responsive caregiving. Caregivers looked positively on children who awakened early and

For peer review only - http://bmjopen.bmj.com/site/about/guidelines.xhtml

quickly, so messages also emphasise the positive effects of caregivers' responsive engagement with children on the timing and speed of awakening. Radio messages also respond to local beliefs which might hinder the practice of ECD behaviours, such as perceptions of the limited impact of responsive interactions on babies in the first months of life, by highlighting the benefits of engaging with babies from birth using terms and examples that are salient to the target population. The integration of formative research makes program messages more relevant and familiar to the target audience, an aspect of ECD intervention development that is important ethically and also optimises program efficacy [10,19,20,33,34].

Parents' aspirations for their children's cognitive and social development and future success also aligned with the benefits of responsive ECD interactions. For example, caregivers said they wanted their children to be articulate and confident in front of strangers, in a classroom and within the community. Success at school was a high priority. Caregivers said talking to children from a young age would, therefore, help them "have the right words", be successful at school and, later in life, be an involved and respected community members. Play was also viewed positively: children who played would do better at school because they would be more "awake", outgoing and curious as active learners. Radio messages, therefore, encourage play from birth but with more caregiver-child and child-led interactions than formative research revealed were occurring. Caregivers are encouraged to play during daily tasks such as bathing given the time constraints many families said they faced. While we agree with authors who critique assumptions that populations across all socio-cultural contexts have the same educational and aspirational goals [14,17,35,40], our research affirms that many parents in this context aspire for their children to have academic success leading to professional careers. In addition, interdisciplinary evidence suggests that impending changes in population demographics, climate, and economics, especially in sub-Saharan Africa, will demand that more individuals are prepared to enter the formal workforce [33,41] making scholastic success relevant to wider populations.

Our study supports the prioritisation of formative research which explores parental ethnotheories in non-Western contexts, including perceptions of if, how and when young children learn and if and how "standard" ECD behaviours and activities fit into context-specific parental and communal aspirations for children. Findings from this process should be legitimised in their own right, not necessarily compared to Western ECD standards to deduce what is "lacking" or what needs to be "fixed" [18], and integrated into intervention development and implementation. Our research and implementation teams prioritised intentional space for iterative reflexivity, which we believe greatly aided our overall adaptation process and made for a more equitable research and implementation experience. We have also continued to collect qualitative data throughout the campaign to feedback into the intervention design, reflecting the need for ongoing contextually sensitive adaptation and improvement [43].

Ethnographic research such as integration into households of the target population for an extended period of time should also be part of the formative research process whenever possible [17]. This type of research could reveal more nuanced information relevant to ECD including the nature of caregiver-child interactions, play, play things, and local games, songs, and poems which existing programming often does not discover or ignores [34,35,44]. Unfortunately, due to security concerns, we were unable to complete ethnographic research as part of this study which could have limited the breadth and depth of our data.

For peer review only - http://bmjopen.bmj.com/site/about/guidelines.xhtml

A limitation of the TIPs methodology in particular is that participants easily identify "desirable" behaviours. Therefore, there is a risk of social desirability bias in participant responses related to current practices and suggested behaviours [45]. We countered this risk during data collection by emphasising our desire to learn about all participant experiences, normalising challenges participants might experience and focused probing. Finally, our findings are particular to a specific research context and are not necessarily generalizable.

Understanding the early childhood norms, beliefs and practices of a local population can reveal important socio-cultural differences between assumptions underlying different ECD curricula and the contexts in which they are implemented. Acknowledging and integrating these differences during program planning and implementation could help achieve more equitable child development and, subsequently, social, health and economic outcomes globally.

Acknowledgements

Sincere thanks to the caregivers who took time out of their days to participate in this study and local authorities and leaders for their collaboration in facilitating access to their communities. Without them, these important perspectives would not be available to the wider scientific community. We would also like to thank the data collectors for their dedication and quality contributions to this study under demanding field circumstances.

Funding

The authors report no conflict of interest. This research was funded by the Wellcome Trust (grant numbers 215492/Z/19/Z [LSHTM] 215492/A/19/Z [DMI]) under the *Scaling Up Nurturing Care, a Radio Intervention to Stimulate Early Childhood Development* (SUNRISE) project.

Competing interests

The authors have no competing interests to declare.

For peer review only

References

1 Black MM, Walker SP, Fernald LCH, *et al.* Early childhood development coming of age: science through the life course. *The Lancet* 2017;**389**:77–90. doi:10.1016/S0140-6736(16)31389-7

2 Holt RL, Mikati MA. Care for Child Development: Basic Science Rationale and Effects of Interventions. *Pediatr Neurol* 2011;**44**:239–53. doi:10.1016/j.pediatrneurol.2010.11.009

3 Garcia MH, Pence A, Evans JL. *Africa's Future, Africa's Challenge : Early Childhood Care and Development in Sub-Saharan Africa*. Herndon, UNITED STATES: : World Bank Publications 2008.

4 Walker SP, Chang SM, Vera-Hernández M, *et al.* Early Childhood Stimulation Benefits Adult Competence and Reduces Violent Behavior. *Pediatrics* 2011;**127**:849–57. doi:10.1542/peds.2010-2231

5 Jeong J, Franchett EE, Oliveira CVR de, *et al.* Parenting interventions to promote early child development in the first three years of life: A global systematic review and meta-analysis. *PLOS Med* 2021;**18**:e1003602. doi:10.1371/journal.pmed.1003602

6 WHO. Improving early childhood development: WHO ECD guideline. 2020. https://www.who.int/publications-detail-redirect/97892400020986 (accessed 16 Jan 2023).

7 Lu C, Cuartas J, Fink G, *et al.* Inequalities in early childhood care and development in low/middle-income countries: 2010–2018. *BMJ Glob Health* 2020;**5**:e002314. doi:10.1136/bmjgh-2020-002314

8 Kohli-Lynch M, Ponce Hardy V, Bernal Salazar R, *et al.* Human resources and curricula content for early child development implementation: multicountry mixed methods evaluation. *BMJ Open* 2020;**10**:e032134. doi:10.1136/bmjopen-2019-032134

9 Gladstone M, Phuka J, Mirdamadi S, *et al.* The care, stimulation and nutrition of children from 0-2 in Malawi—Perspectives from caregivers; 'Who's holding the baby?' *PLOS ONE* 2018;**13**:e0199757. doi:10.1371/journal.pone.0199757

For peer review only - http://bmjopen.bmj.com/site/about/guidelines.xhtml
For peer review only

10 Bentley ME, Johnson SL, Wasser H, *et al.* Formative research methods for designing culturally appropriate, integrated child nutrition and development interventions: an overview. *Ann N Y Acad Sci* 2014;**1308**:54–67. doi:10.1111/nyas.12290

11 Lingam R, Gupta P, Zafar S, *et al.* Understanding care and feeding practices: building blocks for a sustainable intervention in India and Pakistan. *Ann N Y Acad Sci* 2014;**1308**:204–17. doi:10.1111/nyas.12326

12 Greenfield, P and Keller H. P. Cultural Psychology. In: *Encyclopedia of Applied Psychology*. 2004. 545–53.https://www.sciencedirect.com/science/article/pii/B0126574103001951

13 Tsamaase M, Harkness S, Super CM. Grandmothers' Developmental Expectations for Early Childhood in Botswana. *New Dir Child Adolesc Dev* 2020;**2020**.

14 Ng'asike JT. African Early Childhood Development Curriculum and Pedagogy for Turkana Nomadic Pastoralist Communities of Kenya. *New Dir Child Adolesc Dev* 2014;**2014**:43–60. doi:10.1002/cad.20072

15 LeVine RA, New RS. *Anthropology and Child Development: A Cross-Cultural Reader*. John Wiley & Sons 2008.

16 Weber A, Fernald A, Diop Y. When Cultural Norms Discourage Talking to Babies: Effectiveness of a Parenting Program in Rural Senegal. *Child Dev* 2017;**88**:1513–26. doi:10.1111/cdev.12882

17 Morelli G, Quinn N, Chaudhary N, *et al.* Ethical Challenges of Parenting Interventions in Low- to Middle-Income Countries. *J Cross-Cult Psychol* 2018;**49**:5–24. doi:10.1177/0022022117746241

18 Bame Nsamenang A. (Mis)Understanding ECD in Africa: The force of local and global motives | Research Connections. 2008. http://www-wds.worldbank.org/external/default/WDSContentServer/WDSP/IB/2008/03/05/000333038_20080305054902/Rendered/PDF/427000PUB0Afri1sclosed0Feb025020081.pdf (accessed 26 Jan 2023).

19 Kumar V, Kumar A, Ghosh AK, *et al.* Enculturating science: Community-centric design of behavior change interactions for accelerating health impact. *Semin Perinatol* 2015;**39**:393–415. doi:10.1053/j.semperi.2015.06.010

20 Modica V Sarah;Ajmera, Maya;Dunning. Culturally Adapted Models of Early Childhood Education. *YC Young Child* 2010;**65**.

21 Hollowell J, Belem M, Swigart T, *et al.* Age-related patterns of early childhood development practices amongst rural families in Burkina Faso: findings from a nationwide survey of mothers of children aged 0-3 years. *Glob Health Action* 2020;**13**:1772560. doi:10.1080/16549716.2020.1772560

22 Hollowell J, Dumbaugh M, Belem M, *et al.* 'Grandmother, aren't you going to sing for us?' Current childcare practices and caregivers' perceptions of and receptivity to early childhood development activities in rural Burkina Faso. *BMJ Glob Health* 2019;**4**:e001233. doi:10.1136/bmjgh-2018-001233

23 Jeong J, Pitchik HO, Yousafzai AK. Stimulation Interventions and Parenting in Low- and Middle-Income Countries: A Meta-analysis. *Pediatrics* 2018;**141**:e20173510. doi:10.1542/peds.2017-3510

For peer review only - http://bmjopen.bmj.com/site/about/guidelines.xhtml

24 Nair S, Chandramohan S, Sundaravathanam N, *et al.* Father Involvement in Early Childhood Care: Insights From a MEL System in a Behavior Change Intervention Among Rural Indian Parents. *Front Public Health* 2020;**8**.https://www.frontiersin.org/articles/10.3389/fpubh.2020.00516 (accessed 11 Aug 2022).

25 Rothstein JD, Buckland AJ, Gagnier K, *et al.* Assessing the play and learning environments of children under two years in peri-urban Lima, Peru: a formative research study. *BMC Public Health* 2021;**21**:108. doi:10.1186/s12889-020-10119-3

26 Zhang D, Jin L, Liang D, *et al.* Assessing Feasibility of an Early Childhood Intervention Using Mobile Phones Among Low-Income Mothers of Newborns: Qualitative Interview Study. *JMIR Form Res* 2020;**4**:e17179. doi:10.2196/17179

27 Strachan D, Kabwijamu L, Okuga M, *et al.* Shifting the discourse from survive to thrive: a qualitative exploration of beliefs, actions and priorities for early childhood development in Uganda. *J Br Acad* 2020;**8s2**:41–70. doi:10.5871/jba/008s2.041

28 Singla DR, Kumbakumba E, Aboud FE. Effects of a parenting intervention to address maternal psychological wellbeing and child development and growth in rural Uganda: a community-based, cluster-randomised trial. *Lancet Glob Health* 2015;**3**:e458–69. doi:10.1016/S2214-109X(15)00099-6

29 Mebrahtu H, Simms V, Mupambireyi Z, *et al.* Effects of parenting classes and economic strengthening for caregivers on the cognition of HIV-exposed infants: a pragmatic cluster randomised controlled trial in rural Zimbabwe. *BMJ Glob Health* 2019;**4**:e001651. doi:10.1136/bmjgh-2019-001651

30 Barnhart DA, Farrar J, Murray SM, *et al.* Lay-worker Delivered Home Visiting Promotes Early Childhood Development and Reduces Violence in Rwanda: A Randomized Pilot. *J Child Fam Stud* 2020;**29**:1804–17. doi:10.1007/s10826-020-01709-1

31 Ministry of Health Rwanda, UNICEF Rwanda. Knowledge, Attitudes and Practices Assessment on Early Nurturing of Children Report. 2014. https://silo.tips/queue/knowledge-attitudes-practices?&queue_id=-1&v=1680203477&u=NzMuMTg2LjEzOC4yMjg=

32 The Manoff Group. Trials of Improved Practices (TIPS): Giving Participants a Voice in Program Design. 2005.https://www.behaviourchange.net/document/52-trials-of-improved-practices-tips-giving-participants-a-voice-in-program-design

33 Weber AM, Diop Y, Gillespie D, *et al.* Africa is not a museum: the ethics of encouraging new parenting practices in rural communities in low-income and middle-income countries. *BMJ Glob Health* 2021;**6**:e006218. doi:10.1136/bmjgh-2021-006218

34 Serpell R, Nsamenang AB. Locally relevant and quality ECCE programmes: Implications of research on indigenous African child development and socialization. 2014.https://unesdoc.unesco.org/ark:/48223/pf0000226564

35 Nsamenang, A. Bame. In a globalised world, local culture must be the anchor of identity. *Early Child Matters* 2014.

For peer review only - http://bmjopen.bmj.com/site/about/guidelines.xhtml

36 Prochner L, Kabiru M. ECD in Africa: A historical perspective. 2008.https://documents1.worldbank.org/curated/en/135791468211777082/pdf/427000PUB0Afri1sclosed0Feb025020081.pdf

37 Feldman HM. How Young Children Learn Language and Speech. *Pediatr Rev* 2019;**40**:398–411. doi:10.1542/pir.2017-0325

38 Murray L, Cooper P, Arteche A, *et al.* Randomized controlled trial of the effect of a home visiting intervention on infant cognitive development in peri-urban South Africa. *Dev Med Child Neurol* 2015;**58**:270–6. doi:10.1111/dmcn.12873

39 London School of Hygiene and Tropical Medicine. SUNRISE Cluster RCT: Scaling Up Nurturing Care, a Radio Intervention to Stimulate Early Child Development in Burkina Faso. clinicaltrials.gov 2022. https://clinicaltrials.gov/ct2/show/NCT05335395 (accessed 9 Jan 2023).

40 Serpell R. Africentric engagement with child development theory and education: the legacy of Bame Nsamenang. *Int J Behav Dev* 2019;**2019**:3–4.

41 Collier P, Conway G, Venables T. Climate change and Africa. *Oxf Rev Econ Policy* 2008;**24**:337–53.

42 Marfo K, Biersterker L, Sagnia J, *et al.* Responding to the challenge of meeting the needs of children under 3 in Africa. *Afr Future Afr Chall* 2008;:201–26.

43 Chambers DA, Glasgow RE, Stange KC. The dynamic sustainability framework: addressing the paradox of sustainment amid ongoing change. *Implement Sci IS* 2013;**8**:117. doi:10.1186/1748-5908-8-117

44 Lanyasunya AR, Lesolayia MS. *The El-Barta Child and Family Project--Community Based Early Child Care and Development Programme: An Integrated Approach. Working Papers in Early Childhood Development*. Bernard van Leer Foundation, P 2001. https://eric.ed.gov/?id=ED460778 (accessed 7 Sep 2022).

45 Bergen N, Labonté R. "Everything Is Perfect, and We Have No Problems": Detecting and Limiting Social Desirability Bias in Qualitative Research. *Qual Health Res* 2020;**30**:783–92. doi:10.1177/1049732319889354

For peer review only - http://bmjopen.bmj.com/site/about/guidelines.xhtml

Supplemental material

'Children awaken by playing': A qualitative exploration of caregivers' perceptions of young children's learning and early childhood development in rural Burkina Faso

Audio clips played for focus group discussion participants

→ *The following text was audio recorded in French, Bissa and Dioula and played for participants in Focus Group Discussions. Participants were then asked for their reactions to the information shared in the recordings.*

Clip 1: The basics and benefits of early childhood development
Children are curious from the start of their lives. They want to learn and understand the world. During the first five years of life, a child's brain grows faster than at any other time. So anything you say and do can help build a child's brain. Children's early experiences determine their brain development and give them the foundation for success in life. Talking and playing with the child from birth will make him active and intelligent.

Clip 2: Talk
Your children get it before they start talking. When they babble and twitter, they are looking to communicate or 'talk' in their own way with you. Talk to them often and imitate their sounds and expressions to see how they react - each of you can speak in turn!

Your baby loves the sound of your voice. Talk to your baby about everything you do during the day. Describe what you hear, see and smell around you. Point to objects and people and name them. This will facilitate their learning. You can talk to your baby during the bath, while you are dressing her, during meals, when you breastfeed her. You can also do this during household chores (such as cooking, cleaning, and laundry), or when you're sitting with your family and before bedtime. Tell your family to talk to the baby often, too. The more the baby is exposed to language (even before he can speak), the more words he will learn!

Clip 3: Play
It is through play that children learn about the world around them. Give your child your full attention for at least 15 minutes a day. Encourage him to look at your face or at the colored objects around you such as a picture, a plate or a mug.

When your baby gets older, help him discover the world around him by giving him items to hold in his hand to play. Look for safe objects or toys that he can hold in his hands and that he can shake or roll (such as a lime, orange, or any other round object in the house). You can also put beans in a small container with a lid that the baby can use and shake like a rattle. Have fun with him by hiding your face

behind your hands and making him laugh. This will help her understand that things don't go away even if they are hidden.

Take part in your child's play and exploration whenever you can. That way when he gets stuck or gets frustrated, you can help him kindly.

Clip 4: Encouragement and praise

Encouraging your child, at any age, shows him that you are happy with what he has done and it will make him want to do it again. Your child wants to know that you are happy with what he has done and the effort he has put in. Therefore, he will learn faster when you encourage him to try new things. It's always better to encourage your child to do the things you want them to do rather than constantly telling them not to do the things you don't want them to do! So encourage and praise him rather than saying negative words like “bad” or “don't do that.

“Instead, say “well done” and “well done!”, clap or smile when he does something that you like or when he is learning something new. Even babies who have not yet learned to speak are encouraged with positive words. So encourage and praise your baby when he tries to do new things (if he picks up objects or holds them in his hand, if he claps with his hands or tries to stand unaided). Use hugs, kisses and loving words with your child every day. Your child will then be brought in more often to do the activities you want him to do.

COREQ (COnsolidated criteria for REporting Qualitative research) Checklist

A checklist of items that should be included in reports of qualitative research. You must report the page number in your manuscript where you consider each of the items listed in this checklist. If you have not included this information, either revise your manuscript accordingly before submitting or note N/A.

Topic	Item No.	Guide Questions/Description	Reported on Page No.
**Domain 1: Research team and reflexivity**			
Personal characteristics			
Interviewer/facilitator	1	Which author/s conducted the interview or focus group?	
Credentials	2	What were the researcher's credentials? E.g. PhD, MD	
Occupation	3	What was their occupation at the time of the study?	
Gender	4	Was the researcher male or female?	
Experience and training	5	What experience or training did the researcher have?	
Relationship with participants			
Relationship established	6	Was a relationship established prior to study commencement?	
Participant knowledge of the interviewer	7	What did the participants know about the researcher? e.g. personal goals, reasons for doing the research	
Interviewer characteristics	8	What characteristics were reported about the inter viewer/facilitator? e.g. Bias, assumptions, reasons and interests in the research topic	
**Domain 2: Study design**			
Theoretical framework			
Methodological orientation and Theory	9	What methodological orientation was stated to underpin the study? e.g. grounded theory, discourse analysis, ethnography, phenomenology, content analysis	
Participant selection			
Sampling	10	How were participants selected? e.g. purposive, convenience, consecutive, snowball	
Method of approach	11	How were participants approached? e.g. face-to-face, telephone, mail, email	
Sample size	12	How many participants were in the study?	
Non-participation	13	How many people refused to participate or dropped out? Reasons?	
Setting			
Setting of data collection	14	Where was the data collected? e.g. home, clinic, workplace	
Presence of non-participants	15	Was anyone else present besides the participants and researchers?	
Description of sample	16	What are the important characteristics of the sample? e.g. demographic data, date	
Data collection			
Interview guide	17	Were questions, prompts, guides provided by the authors? Was it pilot tested?	
Repeat interviews	18	Were repeat inter views carried out? If yes, how many?	
Audio/visual recording	19	Did the research use audio or visual recording to collect the data?	
Field notes	20	Were field notes made during and/or after the inter view or focus group?	
Duration	21	What was the duration of the inter views or focus group?	
Data saturation	22	Was data saturation discussed?	
Transcripts returned	23	Were transcripts returned to participants for comment and/or	

For peer review only

Topic	Item No.	Guide Questions/Description	Reported on Page No.
		correction?	
**Domain 3: analysis and findings**			
Data analysis			
Number of data coders	24	How many data coders coded the data?	
Description of the coding tree	25	Did authors provide a description of the coding tree?	
Derivation of themes	26	Were themes identified in advance or derived from the data?	
Software	27	What software, if applicable, was used to manage the data?	
Participant checking	28	Did participants provide feedback on the findings?	
Reporting			
Quotations presented	29	Were participant quotations presented to illustrate the themes/findings? Was each quotation identified? e.g. participant number	
Data and findings consistent	30	Was there consistency between the data presented and the findings?	
Clarity of major themes	31	Were major themes clearly presented in the findings?	
Clarity of minor themes	32	Is there a description of diverse cases or discussion of minor themes?	

Developed from: Tong A, Sainsbury P, Craig J. Consolidated criteria for reporting qualitative research (COREQ): a 32-item checklist for interviews and focus groups. *International Journal for Quality in Health Care*. 2007. Volume 19, Number 6: pp. 349 – 357

Once you have completed this checklist, please save a copy and upload it as part of your submission. DO NOT include this checklist as part of the main manuscript document. It must be uploaded as a separate file.

For peer review only

BMJ Open

'Children awaken by playing': A qualitative exploration of caregivers' norms, beliefs and practices related to young children's learning and early childhood development in rural Burkina Faso

Journal:	*BMJ Open*
Manuscript ID	bmjopen-2023-075675.R1
Article Type:	Original research
Date Submitted by the Author:	05-Sep-2023
Complete List of Authors:	Dumbaugh, Mari; University College London, Institute for Global Health; Insight Impact Consulting<br>Belem, Mireille; Develpment Media International, Research<br>Kousse, Sylvain; Development Media International, Research<br>Ouoba, Patricia; Develpment Media International, Research<br>Sankoudouma, Adama ; Innovations for Poverty Action Francophone West Africa<br>Tchibozo, Achille; Innovations for Poverty Action Francophone West Africa<br>Fearon, Pasco; University of Cambridge, Department of Psychology; University College London, Centre for Family Research, Department of Psychology<br>Hollowell, Jennifer ; Development Media International Associates CIC,<br>Hill, Z; University College London Research Department of Epidemiology and Public Health, Institute for Global Health
**Primary Subject Heading**:	Global health
Secondary Subject Heading:	Qualitative research
Keywords:	Health Equity, Community child health < PAEDIATRICS, Health Education, QUALITATIVE RESEARCH

SCHOLARONE™
Manuscripts

I, the Submitting Author has the right to grant and does grant on behalf of all authors of the Work (as defined in the below author licence), an exclusive licence and/or a non-exclusive licence for contributions from authors who are: i) UK Crown employees; ii) where BMJ has agreed a CC-BY licence shall apply, and/or iii) in accordance with the terms applicable for US Federal Government officers or employees acting as part of their official duties; on a worldwide, perpetual, irrevocable, royalty-free basis to BMJ Publishing Group Ltd ("BMJ") its licensees and where the relevant Journal is co-owned by BMJ to the co-owners of the Journal, to publish the Work in this journal and any other BMJ products and to exploit all rights, as set out in our licence.

The Submitting Author accepts and understands that any supply made under these terms is made by BMJ to the Submitting Author unless you are acting as an employee on behalf of your employer or a postgraduate student of an affiliated institution which is paying any applicable article publishing charge ("APC") for Open Access articles. Where the Submitting Author wishes to make the Work available on an Open Access basis (and intends to pay the relevant APC), the terms of reuse of such Open Access shall be governed by a Creative Commons licence – details of these licences and which Creative Commons licence will apply to this Work are set out in our licence referred to above.

Other than as permitted in any relevant BMJ Author's Self Archiving Policies, I confirm this Work has not been accepted for publication elsewhere, is not being considered for publication elsewhere and does not duplicate material already published. I confirm all authors consent to publication of this Work and authorise the granting of this licence.

For peer review only

'*Children awaken by playing'*: A qualitative exploration of caregivers' norms, beliefs and practices related to young children's learning and early childhood development in rural Burkina Faso

Mari Dumbaugh[1,2], Mireille Belem[3], Sylvain Kousse[3], Patricia Ouoba[3], Adama Sankoudouma[4], Achille Mignondo Tchibozo[4], Pasco Fearon[5,6], Jennifer Hollowell[7], Zelee Hill[1] and the SUNRISE team

1. Institute for Global Health, University College London, UK
2. Insight Impact Consulting, Chicago, USA
3. Development Media International Burkina Faso
4. Innovations for Poverty Action, Francophone West Africa, Burkina Faso
5. Centre for Family Research, Department of Psychology, University of Cambridge, UK
6. Research Department of Clinical, Educational and Health Psychology, University College London, UK
7. Development Media International, London, UK

Corresponding author:
Dr Zelee Hill
z.hill@ucl.ac.uk

Institute for Global Health
University College London
30 Guilford Street
London, England
WC1N 1EH

For peer review only - http://bmjopen.bmj.com/site/about/guidelines.xhtml

'*Children awaken by playing'*: A qualitative exploration of caregivers' norms, beliefs and practices related to young children's learning and early childhood development in rural Burkina Faso

Keywords: Early childhood development; early learning; formative research; qualitative research; cultural adaptation; Burkina Faso; SUNRISE

Abstract

Introduction

Evidence suggests that responsive caregiving and early learning activities positively impact developmental outcomes, with positive effects throughout the life-course. ECD interventions should align with local values, beliefs and resources but there has been little research of caregiver beliefs and perspectives on development and learning, especially in sub-Saharan Africa. This qualitative study explored norms, beliefs, practices and aspirations around child development of caregivers of young children in rural Burkina Faso.

Methods

We conducted 32 in-depth interviews with mothers and fathers of young children and 24 focus group discussions with mothers, fathers and grandmothers, which included trying behaviours and reporting on experiences. The research informed the development of SUNRISE, an early child development radio intervention.

Results

Caregivers described a process of "awakening", through which children become aware of themselves and the world around them. . Perceptions of the timing of awakening varied, but the ability to learn was thought to increase as children became older and more awake. Consequently, talking and playing with babies and younger children was perceived to have little developmental impact. Caregivers said children's interactions with them, alongside God-given intelligence, was believed to impact later behaviour and development. Caregivers felt their role in helping their children achieve later in life was to pay for education, save money, provide advice and be good role models – rather than through interaction and learning activities. Caregivers who trialled interaction and learning activities reported positive experiences for themselves and their child, but interactions were often care-giver led and directive and play was often physical. Key barriers to carrying out the behaviours were poverty and a lack of time.

Conclusions

Exploring early childhood beliefs and practices can reveal important socio-cultural beliefs which, if incorporated into program planning and implementation, could help achieve more impactful, acceptable and equitable programs.

Strengths and limitations of this study

- The use of different qualitative methods, including *Trials of Improved Practices,* allowed us to explore social norms and beliefs of early learning and caregiving as well as how norms and beliefs played out in caregivers' lived experiences.

For peer review only - http://bmjopen.bmj.com/site/about/guidelines.xhtml

- Our South-North research team prioritised intentional space for iterative reflexivity and co-learning, ensuring our research tools, training of data collectors, approaches to data collection and analysis were triangulated and validated from multiple positionalities.
- Logistics prevented us from speaking to caregivers under the age of 18, so our results do not reflect perspectives from this important caregiver group in the Burkinabé context.
- Security concerns limited our ability to incorporate participant-observation into our study which could reveal more nuanced information relevant to early childhood intervention design.

Introduction

Evidence suggests that responsive caregiving and early learning activities positively impact early childhood development (ECD) outcomes, educational achievement and economic opportunities throughout the lifecourse [1–6]. Supporting caregivers to provide responsive care and learning activities during children's early years is considered a key strategy for achieving equitable health and development through platforms such as individualized home visits, group interventions and primary health care contacts [1,5,7].

Parental ethnotheories, or "the shared beliefs about the goals of child development and the socialisation practices that will achieve these goals" [8, p.7], can vary greatly between settings [3,8–15]. Despite progress in developing ECD measures relevant across cultures [16], much of developmental science that informs intervention design originates from research in high-income, Euro-American countries by researchers and with children of the same demographic [10,13,17–19]. There is general consensus that ECD interventions require adaptations for different socio-cultural contexts practices to ensure the development of ethical and effective interventions [13,17,19–24]. A "one size fits all" approach to ECD curricula and interventions risks missing or even damaging important context-specific beliefs, cultural values and social cohesion [10,13,17]. Debates continue, however, as to how to counter research biases and what they mean for programming and measurement [13,17,18,20,25–27].

Formative research has informed the design of ECD interventions in low and middle income countries [12,15,21,22,28–34]. However, few trials in sub-Saharan Africa reported using formative research or local community advisory boards in intervention design, and there has been little research exploring caregiver beliefs and perspectives on development and learning from birth [12,15,22,34–38]. We conducted formative qualitative research in rural Burkina Faso to explore ECD norms, beliefs and practices and aspirations of caregivers of children aged zero to three years. This research informed the development of *Scaling Up Nurturing Care, a Radio Intervention to Stimulate Early Childhood Development* (SUNRISE), running from 2022-2025 and offering caregivers ECD information via radio broadcast to encourage responsive engagement with young children (Trial registration: Clinicaltrials.gov identifier NCT05335395).

Methods

We conducted semi-structured, in-depth interviews (IDIs) with mothers and fathers and focus group discussions (FGDs) with mothers, fathers and grandmothers. FGDs allowed us to explore social norms and beliefs and IDIs to understand how these played out in caregivers' lived experiences. Data were collected in four villages in Balé and Boulgou provinces, with equal numbers of participants sampled from each

For peer review only - http://bmjopen.bmj.com/site/about/guidelines.xhtml

village (see **Table 1**). Villages were safe for research teams and did not have exceptional characteristics such as being located along a major thoroughfare. They had a range of ethnicities to complement previous data we had collected on the country's dominant ethnic group (the Mossi) [15].

Ethics

The Burkina Faso Institute for Health Science Research Ethics Committee and the University College London Research Ethics Committee granted ethical approval for this study.

Sampling & Data collection

Data were collected from March-April 2021. Data collectors and supervisors (women and men) had completed two to four years of undergraduate study and had previous experience collecting qualitative data. They underwent three weeks of training to review methods, ethics, research tools and transcription. We conducted a pilot study in Ouagadougou to finalise the data collection guides. Supervisors were responsible for logistics, recruitment and data quality; data collectors obtained informed consent and conducted IDIs and FGDs.

Supervisors used the same procedure to recruit for IDIs and FGDs: First they introduced the study to village leaders who identified a community informant knowledgeable about the community, but not a member of the village chief's family. The informant, often a community health worker, then accompanied the supervisor to households with eligible children, where supervisors introduced the study and provided an information sheet detailing the study objectives, confidentiality, participants' rights and research team contact numbers. For participants unable to read French, information and consent forms were read aloud with a non-research team member witnessing and co-signing. For those unable to write, a thumb print replaced a signature. Supervisors purposively selecting participants over 18 years old, ensuring that a range of children's ages were represented: zero to three years for IDIs and under 24 months for FGDs as previous research shows caregivers are less likely to engage interactively with this age group in Burkina Faso [15,29].

IDIs were conducted in a private place in participants' homes and explored typical daily routines, parents' perceptions of children's early learning and parents' short and long term aspirations for their children (see Supplemental Appendix 1). Participants were given soap after each IDI to thank them for their time. FGDs were stratified by participant group to allow topics to be discussed from a perspective of similar positionalities. Two FGDs were conducted, in a neutral space away from other community members, with each participant group in each village by a facilitator and note taker.

FGDs used an adapted *Trials of Improved Practice* (TIPS) methodology [15,39]. A first round explored norms and beliefs around early child learning and caregiving (see Supplemental Appendix 1). Then, participants listened to four recordings on the impact of early experiences on development, the importance of talking to children from birth, play as a vehicle for early learning and development and the benefits of frequent praise, encouragement and affection (see Supplemental Appendix 2). Participants shared their reactions to the spots and were invited to practise talking, playing or praising/ encouraging their child at home, and return for a second FGD in one week's time to share their experiences, challenges

For peer review only - http://bmjopen.bmj.com/site/about/guidelines.xhtml

and child, family and community member reactions to the behaviours. Participants received money for transport. Four participants were lost to follow up in the second FGDs (two fathers, one mother, and one grandmother).

Research activities lasted 45-90 minutes, were conducted in local languages (Bissa or Dioula) and audio recorded. Given local gender norms which might have induced response bias between researchers and participants of different genders, data collectors were paired with participants of their same gender.

Table 1. details participant socio-demographics (see also Supplemental Appendix 3). Participants were married or widowed and identified their religion as Muslim, Christian or animist/ traditional. Ten ethnic groups were represented in the sample population, with about half of participants identifying as Bissa. Participants' education levels ranged from none to high school completion. Most educated participants had completed some or all of primary school. The vast majority were farmers.

Table 1. Participant socio-demographics

Research Activity	Participant group	Total number of participants	Participant age range	Range of number of children**	Age ranges of children**		
					0-11 mo	*12-23 mo*	*24+ mo*
In-depth interviews	Mothers	16	20-40	1-7	6	8	2
	Fathers	16	23-59	1-15	8	7	1
*Focus Group Discussions**	Mothers	28	18-36	1-6	13	8	0
	Fathers	26	24-58	1-10	17	9	0
	Grandmothers	27	40-68	1-10	20	7	0

* 5-7 participants per FGD
** For Grandmothers, refers to number/ ages of grandchildren

Data transcription and analysis

Audio data was translated and transcribed into French. To ensure data quality, the first interviews were double transcribed by data collectors in the field and Ouagadougou-based transcriptionists. Senior researchers compared transcriptions for accuracy, giving translators feedback on translations and interviewers feedback to enhance their reflexivity and improve probing. Throughout data collection audio and transcripts were reviewed to ensure accurate translations.

Transcripts were analysed by francophone (MB, MD, SK, PO) and anglophone (ZH, MD) senior researchers. Non-francophone researchers translated transcripts using Google Translate. We used a deductive-inductive approach to content analysis. First, team members read three IDI and three FGD transcripts to

For peer review only - http://bmjopen.bmj.com/site/about/guidelines.xhtml

ensure familiarization with data and themes. A bilingual researcher deductively developed initial code books for each data collection method based on the topics covered in the research guides. Then, a selection of IDIs and FGDs were double coded inductively within these broad themes to develop sub-themes and facilitate standardized coding between team members. The team discussed any variation in coding and resolved differences via consensus discussion. Once coding was standardized and adequately detailed, the remaining transcripts were divided between researchers for analysis. We held frequent meetings to discuss findings, clarify translations, iteratively refine the code books and confirm that saturation was reached within our planned sample size (additional data collection would not have produced new information). Francophone researchers used NVIVO (released March 2020) to complete analysis; the anglophone researcher coded by hand.

Participant and Public Involvement

Participants and/or the public were not involved in the design, conduct, reporting, or dissemination plans of this research. Final transcriptions and findings were not sent to participants, however participants were given contact numbers of research team leads should they want to review data or results.

Results

Analysis resulted in four major themes: perceptions of young children's consciousness; when and how children become conscious and learn; impacts of caregiver-child interactions; and, parents' aspirations for their children. We have noted where differences in results were observed by caregiver role. Caregiver perspectives reported in this paper did not differ by the caregiver's or child's gender (child's gender only collected for IDIs), or between ethnic groups.

Perceptions of young children's consciousness

Caregivers consistently conceptualised the development of child consciousness as a process of "awakening": a child becoming aware of themselves and the world around them, their intellectual development, increasing capacity to understand and act with intelligence, their ability to follow directions, to understand the difference between right and wrong and to act on socio-cultural norms such as greeting people, sharing food with others and bringing visitors water.

> *When a visitor arrives at the house, the child quickly goes to greet the visitor and gets water for him…The child tries to do what [adults] do around him. In doing so, he…[shows he] is truly awake.* (Grandmother; grandchild 4 months)

The process, timing and speed of awakening was unique to each child and influenced by the "God-given" nature of the child, caregivers' behaviours, or a combination of both.

When and how children become conscious and learn

A major finding was caregivers' differing perceptions as to when children began to awaken and learn, varying from the earliest months of life to five years of age and the start of formal schooling. Caregivers said babies were not awake from birth as they were born without knowledge, memory or skills. The few

For peer review only - http://bmjopen.bmj.com/site/about/guidelines.xhtml

caregivers who said that babies were capable of learning new things in their first months cited various signs of early learning: smiling and laughing; "playing" with their hands; crying for a diaper change; following people with their eyes; recognizing people; and acquiring new motor skills.

Perceptions of the timing of children's language comprehension also varied, from the earliest months of life to older than two years. Most caregivers thought that children usually start to comprehend language after about one year old, and show it by repeating and remembering words:

> *At 15 months the child begins to grasp some words and understand them. First he mixes up words, he calls people but … you cannot understand who he is calling. At 16 or 17 months he begins to talk more clearly, he knows how to call his father and mother, and that progresses gradually until he is 20 or 24 months old.* (Father; child 33 months)

Fewer caregivers thought that preverbal children could learn to recognize and engage in forms of communication such as understanding their mother's expressions and tones, recognizing when people were talking or being quiet and comprehending simple instructions from adults.

> *A child knows words even when he is small. Even at five months, if you say something, [the child] hears but he cannot speak…Sometimes when you say certain things, …[the child] starts to cry. He cries because he understood something. [The child] begins…to hear words without you realising it.* (Father; child 11 months)

Caregivers said children learn to talk through interactions such as call and response, imitation, repetition and play with adults and other children.

Talking to young babies in a conversant style was not common because of the perception that babies did not yet understand words and could not respond. Caregivers felt that talking to a preverbal baby was like talking to yourself. FGD participants highlighted this challenge after trying TIPS behaviours in their homes:

> *What was difficult for me was when the child said "ha ha ha!" That means that he wants to talk to us, but we do not understand what he says, so we tell him to stop… and be quiet. As a Bissa saying goes, "A mute person cannot say to his grandmother, 'I want such and such a thing.'"* (Grandmother; grandchild 1 month)

Caregivers also said some community members felt that talking to preverbal babies or playing with children was bizarre or a waste of time, with no real benefit to the young child.

> *I was sitting with my child in the middle of the day and I heard a knock at the door. The person [at the door] asked me who I was talking to, and I said I was talking to my baby....I told her my baby enjoys it when I talk to her … The person replied, "What does this little child know for you to speak [to her]?" I told her [my baby] understands and that's why I'm chatting with her. [My baby] laughs and she appreciates it, and I like it too. The person …[told] me that the [only] reason I am [talking to my baby] is that I don't have a job.* (Mother; child 1 month)

For peer review only - http://bmjopen.bmj.com/site/about/guidelines.xhtml

Despite divergence on when exactly awakening and language comprehension begin, a major finding was that caregivers agreed on the learning process itself. They said young children learn “little by little”, learning more and more quickly as they awaken and are older. Children learnt through observation, recall, imitation and repetition of others; talk and explanations by caregivers; play on their own or with others; being corrected or encouraged; and at school through formal teaching. Talking to verbal children was believed to help children understand and learn words and recognize loved ones.

> *A child who is not used to having people talk to him will be less intelligent. But, a child who has always had people talking to him, that is like school for him and he will do remarkable things. You will see he is truly awakened.* (Grandmother; grandchild 1 month)

Play, or “children’s work”, alone or with peers, was considered particularly important for development and learning as it helped awaken children and “open their spirit”, helped children learn new words, expanded motor skills such as sitting up, clapping hands, crawling, walking and dancing; encouraged independence; made children intelligent by providing new experiences, socialization with others, and taught children new ideas and lessons such as dangerous things to avoid (ie fire); and was an opportunity to practise life tasks through pretend games like cooking, playing shop, washing clothes and farming.

> *Play awakens children. If a child plays, he learns many things... Play also develops his intellectual capacities.* (Mother; child 2 months)

Children usually played independently or with siblings and peers. Play between adults and children was mostly physical such as bouncing up and down, and other forms of play was not widespread, but caregivers who played with their children after FGDs had positive experiences.

Awakening and learning were at once influenceable and predestined. For example, while caregivers said play and talk could contribute to children’s early or quick awakening, they also said these interactions would have limited influence until children were sufficiently awake (usually around two to three years old). Most caregivers also said “God given” or innate intelligence, character traits or circumstances determined children’s development and learning.

> *God plots the future of each child.* (Grandmother; grandchild 4 months)

> *Some kids get better at a game the more they play. There are other children who sit and do not know how to play; everything is in the hands of God.* (Mother; child 21 months)

A child who “lacks blessings”, for example, may be a difficult child or slow to follow instructions. Or, the quality of a woman’s breast milk could positively or negatively impact her children’s developmental achievements.

While recognizing that each child’s developmental path was unique, caregivers had expectations around the timing of developmental milestones, and noticeable delays were cause for concern:

For peer review only

> *Children…do not all have the same growth rhythm. At six months, some are sitting up and crawling…There are other [children] who do not grow quickly. At one year they do not want to walk and it scares you…* (Mother; child 12 months)

Future impacts of caregiver-child interactions

Caregivers believed early experiences could impact a child's later behaviours and development, and that positive caregiver-child interactions were beneficial in the short and long terms.

> *Some women insult their children…, for example, 'Idiot, God will punish you!' But, the child hears what you say without being able to respond, and he grows up with the words spoken to him. So, if we speak to the child using [kind] words, he will grow up with that.* (Mother; child 18 months)

The perceived effects of disciplinary practices demonstrated the same. While caregivers universally agreed that sanctions and discipline were important aspects of child rearing, many also said that too frequent or too harsh discipline could have negative consequences for the child and their relationship with their parents well into adulthood.

The positive, long-term impacts of speaking to children softly, with positive words and encouragement were emphasised:

> *When you scold a child a lot, he gets confused; so you have to speak to him softly…this is when his intelligence will increase.* (Mother; child under 6 months)

FGD participants said children reacted positively to talk, play and praise during the TIPS. Even very young babies reacted to ECD behaviours through direct eye contact, giggling, babbling and moving their arms and legs:

> *When he sees me he looks at me with big eyes and he moves [to show] he understands. When he sees me, he jumps, he laughs and he looks at me; so I know he understands.* (Mother; child 5 months)

Older children's reactions to ECD behaviours tended to be more pronounced. After engaging in ECD behaviours, caregivers said older children responded more positively to them, were more aware of their presence, ran to greet them and asked them to play again. They said children were happier, more awake, had learnt to play or played better than before, increased their understanding and level of communication, had improved attitudes, followed directions, cried less and were more likely to interact and share with others.

> *Before [I tried the ECD practices], …my child…did not want to share with anyone else. But, now, with the little time that I have played with him, he has gotten used to me…His mother bought him peanuts and…I asked him to give his grandmother one and he did. This is how I know he recognizes me now, as his father, thanks to the time I spent with him.* (Father; child 30 months)

For peer review only - http://bmjopen.bmj.com/site/about/guidelines.xhtml

While these tangible changes were positive, the interactions caregivers described tended to be caregiver-led and directive in nature, rather than child-led and responsive to the child's signs and reactions. For example, talking at the child to give directions or monologuing rather than engaging in a conversational exchange with the child, exclusively physical play (ie lifting child in air, tickling) or giving children objects to keep them busy or console them rather than facilitating play with the child.

Parents' aspirations for their children

IDI participants were asked to describe their aspirations for their children. In the short-term, parents wanted happy, healthy children who were successful at school. They wanted their children to be close to them, respectful of elders and visitors, helpful without being told what to do and good at following instructions and advice. They also described desirable character traits: independence, curiosity, politeness, active and alert (especially at school) and playful, as play signalled a healthy and happy child.

In the longer term, parents wished for their grown children to be healthy, continue practising their religion, marry a good person and have children themselves. Parents wanted children to be "better off" than they were financially by acquiring money, land, a house, and animals, and hoped their children would visit them often and be able to support them financially in old age. Many parents wanted their children to learn a trade, or to have a particular profession such as a government worker, health provider, entrepreneur, school teacher, religious teacher, soldier or police.

> *If you put [your child] in school, he can be awakened and study, go farther than [you].* (Father; child 16 months)

Parents also hoped their children would become respected and forgiving community members, as their success as parents would be measured by their adult children's esteem in the community.

> *I would like her to be forgiving…care for others. If you do not have a good heart and you do not care for others, no one will become close with you*. (Father; child 14 months)

Parents said they could help facilitate their children's success by paying for their education, saving money for them, guiding them with good, moral advice and being good role models. But, they rarely mention ECD behaviours as something they could do to facilitate success:

> *It is up to us [parents] to show them, from a young age, what to do and what not to do to succeed in their life. All of this is through advice, if you do not guide your child, he won't succeed in life, and help you [in your old age].* (Mother; child 10 months)

Childrearing in extreme poverty presented parents with incessant challenges. A lack of food, treating household illnesses and injuries, having a baby before the youngest child had grown and maintaining patience with children also added to household difficulties.

> *Raising children, it's not easy - go to the hospital, eat, it's all very complicated for us. They say that [health services] are free, but they don't give you [free] medicines, so you are there suffering with your child. If you do not have money, it is exhausting to be a parent.* (Father; child 8 months)

Parents said the constant need to seek work, money and provide for the household left them with little

For peer review only - http://bmjopen.bmj.com/site/about/guidelines.xhtml

time to practise ECD behaviours with their young children as often as they would like. Women in particular mentioned the effects of their high burden of housework, and some men worked away from home for extended periods of time.

> *My work is too much. During the day I have to prepare [food]; to pray; washing the children. Women [in our community] have a lot of work.* (Mother; child 12 months)

Discussion

This study expands the evidence base documenting ECD norms, beliefs and practices for young children in sub-Saharan Africa, while also contributing to calls for culturally-relevant adaptations of ECD curricula and programming for non-Western contexts [9,10,13,17,19,40]. Evidence demonstrates the positive effects of a standard set of ECD behaviours across different cultural contexts [1,4,27,41], but authors argue that "imported" ECD curricula "cannot fully meet the needs of African families" [42, p. 130], as they will not reflect fundamental cultural and conceptual differences between Western parenting beliefs and practises and the diverse parental ethnotheories across the Continent [13,14,17].

The SUNRISE intervention design team, a mix of local and UK-based mass media communication experts, used our findings to highlight the benefits of ECD behaviours in alignment with local values, beliefs, practises and aspirations in a rural, sub-Saharan Africa context. Following the *Saturation+* approach to behaviour change, these briefs were scripted into dramatic sketches, then locally produced and broadcast via radio in seven Burkinabé languages multiple times a day [43]. Local radio presenters were also trained to host live, call-in talk shows on ECD-related topics [44].

SUNRISE messages encourage responsive interactions with children from birth by integrating and validating local normative perceptions and beliefs, using terms and scenarios that are salient to the population in rural Burkina Faso. For example, caregivers' belief that ECD behaviours would have a positive impact on their children's development in the short- and long-term became a central SUNRISE message. The concept of "awakening," which echoes descriptions of child development in other similar settings [14], defined most discussions about ECD in our study. Therefore, SUNRISE broadcasts use local perceptions of "awakening" to explain the principles of ECD and encourage responsive caregiving. Caregivers looked positively on children who awakened early and quickly, so messages emphasise the positive effects of caregivers' responsive engagement with children on the timing and speed of awakening. Formative research revealed caregiver-child interactions were directive in nature, so radio messages encourage child-led play from birth. Radio messages also integrate local beliefs which might hinder the practice of ECD behaviours. Given the poverty-induced time constraints many families faced, broadcasts encourage play during daily tasks such as bathing. Messages also respond to the perceived limited impact of responsive interactions on babies in the first months of life, a belief also reported elsewhere in Burkina Faso and Senegal [12,29], and that it is "crazy" or a waste of time for adults to play with babies.

Parents' aspirations for their children's development and future success also aligned with documented benefits of responsive ECD interactions. Caregivers said they wanted their children to be articulate and

For peer review only - http://bmjopen.bmj.com/site/about/guidelines.xhtml

confident in front of strangers and within the community, and success at school was a high priority. After practicing ECD behaviours at home, caregivers said talking to and playing with children from a young age would help them “have the right words”, be successful at school and, later in life, be involved and respected community members. While we agree with authors who critique assumptions that populations across all socio-cultural contexts share the same educational and aspirational goals [10,13,45,46], our research affirmed that many parents in this context aspire for their children to have academic success leading to professional careers. SUNRISE messages, therefore, highlight success at school as one of many benefits of ECD behaviours.

The above examples demonstrate the importance of formative research which explores parental ethnotheories in non-Western contexts and if and how “standard” ECD behaviours fit into context-specific parental and communal beliefs, practices and aspirations for children. Findings from this process should be legitimised in their own right, not necessarily compared to Western ECD standards to deduce what is “lacking” or what needs to be “fixed” [14], and integrated into intervention development and implementation. This makes program messages more relevant and familiar to the target audience, an aspect of ECD intervention development that is important ethically, and also optimises program efficacy [17,23,40,47,48]. Methodologically, our South-North research and implementation teams prioritised intentional space for iterative reflexivity and co-learning, which greatly aided our overall adaptation process. We have also continued to collect qualitative data throughout the campaign, reflecting the need for and benefits of ongoing contextually sensitive adaptation and improvement [49].

Ethnographic research such as participant-observation [13] could reveal more nuanced information relevant to ECD which existing programming often does not discover or ignores [17,45,50], but security concerns meant that this was not possible as part of this study. Logistical challenges also prevented us from speaking to individuals younger than 18 years old, an important limitation given high rates of adolescent pregnancy in Burkina Faso [51]. A limitation of the TIPs methodology in particular is that participants can easily identify “desirable” behaviours with the risk of social desirability bias related to current practices and suggested behaviours [52]. We countered this risk by emphasising our desire to learn about all participant experiences, normalising potential challenges and focused probing. In future, the participation of local populations at every stage of formative research and intervention co-design [53] could be key in realizing the highest level of ethical, culturally adapted, effective ECD programming. Finally, our findings are particular to a specific research context and are not necessarily generalizable.

Understanding the early childhood norms, beliefs and practices of a local population can reveal important socio-cultural differences between assumptions underlying different ECD curricula and the contexts in which they are implemented. Acknowledging and integrating these differences during program planning and implementation could help achieve more equitable child development and, subsequently, social, health and economic outcomes globally.

Acknowledgements

Sincere thanks to the caregivers who took time out of their days to participate in this study and local authorities and leaders for their collaboration in facilitating access to their communities. Without them,

For peer review only

For peer review only - http://bmjopen.bmj.com/site/about/guidelines.xhtml

these important perspectives would not be available to the wider scientific community. We would also like to thank the data collectors for their dedication and quality contributions to this study under demanding field circumstances.

Authorship statement

The following are members of SUNRISE (Scaling Up Nurturing care, a Radio Intervention to Stimulate Early child development): Seeba Amenga-Etego (Ghana), Pare Balary Touba (Burkina Faso), Mireille Belem (Burkina Faso), Radha Chakraborty (UK), Abbie Clare (UK), Cecily Cocks (UK), Sabin Dandjinou (Burkina Faso), Mari Dumbaugh (USA), Pasco Fearon (UK), Roy Head (UK), Zelee Hill (UK), Jennifer Hollowell (UK), Bassirou Kagone (Burkina Faso), Betty Kirkwood (UK), Sylvain Kousse (Burkina Faso), Alexander Manu (UK), Achille Mignondo Tchibozo (Burkina Faso), Joanna Murray (UK), Guikierba Namoano (Burkina Faso), Patricia Ouoba (Burkina Faso), Tom Palmer (UK), Reetabrata Roy (India), Adama Sankoudouma (Burkina Faso), Jolene Skordis (UK), Achille Tchibozo (Burkina Faso).

Contributorship statement

PF, JH and ZH designed the formative research reported on in this paper. MD and ZH designed the data collection tools with input from MB, PF, JH, SK and PO. MD and ZH designed data collector training and MB, SK, PO, AS and AT facilitated the training. AS and AT supervised data collection and monitored the quality of data, data transcription and data translation. MB, MD, ZH, SK and PO analysed data. MD and ZH drafted and revised the paper, with significant input from MB, PF, JH, SK, PO, AS and AT. ZH is guarantor of the study. All members of SUNRISE have made significant intellectual contributions to the SUNRISE campaign and trial design and execution, of which the formative research reported on in this paper was a part.

Competing interests

The authors have no competing interests to declare.

Funding

The authors report no conflict of interest. This research was funded by the Wellcome Trust (grant numbers 215492/Z/19/Z [LSHTM] 215492/A/19/Z [DMI]) under the *Scaling Up Nurturing Care, a Radio Intervention to Stimulate Early Childhood Development* (SUNRISE) project.

Data sharing statement

De-identified transcripts of semi-structured, in-depth interviews and focus group discussions are available from the corresponding author (z.hill@ucl.ac.uk) upon reasonable request. Data collection tools are included as supplementary material to this publication.

For peer review only - http://bmjopen.bmj.com/site/about/guidelines.xhtml

References

1 Black MM, Walker SP, Fernald LCH, *et al.* Early childhood development coming of age: science through the life course. *The Lancet* 2017;**389**:77–90. doi:10.1016/S0140-6736(16)31389-7

2 Holt RL, Mikati MA. Care for Child Development: Basic Science Rationale and Effects of Interventions. *Pediatr Neurol* 2011;**44**:239–53. doi:10.1016/j.pediatrneurol.2010.11.009

3 Garcia MH, Pence A, Evans JL. *Africa's Future, Africa's Challenge : Early Childhood Care and Development in Sub-Saharan Africa*. Herndon, UNITED STATES: : World Bank Publications 2008.

4 Walker SP, Chang SM, Vera-Hernández M, *et al.* Early Childhood Stimulation Benefits Adult Competence and Reduces Violent Behavior. *Pediatrics* 2011;**127**:849–57. doi:10.1542/peds.2010-2231

5 Jeong J, Franchett EE, Oliveira CVR de, *et al.* Parenting interventions to promote early child development in the first three years of life: A global systematic review and meta-analysis. *PLOS Med* 2021;**18**:e1003602. doi:10.1371/journal.pmed.1003602

6 Black MM, Behrman JR, Daelmans B, *et al.* The principles of Nurturing Care promote human capital and mitigate adversities from preconception through adolescence. *BMJ Glob Health* 2021;**6**:e004436. doi:10.1136/bmjgh-2020-004436

7 WHO. Improving early childhood development: WHO ECD guideline. 2020. https://www.who.int/publications-detail-redirect/97892400020986 (accessed 16 Jan 2023).

8 Greenfield, P and Keller H. P. Cultural psychology. In: Spielberger CD, ed. *Encyclopedia of applied psychology*. Elsevier Science & Technology 2004. http://proxy.cc.uic.edu/login?url=https://search.credoreference.com/content/entry/estappliedpsyc/cultural_psychology/0?institutionId=1361 . (accessed 3 Aug 2023).

9 Tsamaase M, Harkness S, Super CM. Grandmothers' Developmental Expectations for Early Childhood in Botswana. *New Dir Child Adolesc Dev* 2020;**2020**.

10 Ng'asike JT. African Early Childhood Development Curriculum and Pedagogy for Turkana Nomadic Pastoralist Communities of Kenya. *New Dir Child Adolesc Dev* 2014;**2014**:43–60. doi:10.1002/cad.20072

11 LeVine RA, New RS. *Anthropology and Child Development: A Cross-Cultural Reader*. John Wiley & Sons 2008.

12 Weber A, Fernald A, Diop Y. When Cultural Norms Discourage Talking to Babies: Effectiveness of a Parenting Program in Rural Senegal. *Child Dev* 2017;**88**:1513–26. doi:10.1111/cdev.12882

13 Morelli G, Quinn N, Chaudhary N, *et al.* Ethical Challenges of Parenting Interventions in Low- to Middle-Income Countries. *J Cross-Cult Psychol* 2018;**49**:5–24. doi:10.1177/0022022117746241

For peer review only

For peer review only - http://bmjopen.bmj.com/site/about/guidelines.xhtml

14 Bame Nsamenang A. (Mis)Understanding ECD in Africa: The force of local and global motives | Research Connections. 2008. http://www-wds.worldbank.org/external/default/WDSContentServer/WDSP/IB/2008/03/05/000333038_20080305054902/Rendered/PDF/427000PUB0Afri1sclosed0Feb025020081.pdf (accessed 26 Jan 2023).

15 Hollowell J, Dumbaugh M, Belem M, *et al.* ‘Grandmother, aren’t you going to sing for us?’ Current childcare practices and caregivers’ perceptions of and receptivity to early childhood development activities in rural Burkina Faso. *BMJ Glob Health* 2019;**4**:e001233. doi:10.1136/bmjgh-2018-001233

16 McCray G, McCoy D, Kariger P, *et al.* The creation of the Global Scales for Early Development (GSED) for children aged 0–3 years: combining subject matter expert judgements with big data. *BMJ Glob Health* 2023;**8**:e009827. doi:10.1136/bmjgh-2022-009827

17 Serpell R, Nsamenang AB. Locally relevant and quality ECCE programmes: Implications of research on indigenous African child development and socialization. 2014.https://unesdoc.unesco.org/ark:/48223/pf0000226564

18 Scheidecker G, Chaudhary N, Oppong S, *et al.* Different is not deficient: respecting diversity in early childhood development. *Lancet Child Adolesc Health* 2022;**6**:e24–5. doi:10.1016/S2352-4642(22)00277-2

19 McCoy DC, Wolf S, Tsinigo E. Improving Early Childhood Development on a Global Scale: Best Practices for Intervention. *JAMA Pediatr* 2022;**176**:337–8. doi:10.1001/jamapediatrics.2021.5351

20 Bame Nsamenang A. Human ontogenesis: An indigenous African view on development and intelligence. *Int J Psychol* 2006;**41**:293–7. doi:10.1080/00207590544000077

21 Kohli-Lynch M, Ponce Hardy V, Bernal Salazar R, *et al.* Human resources and curricula content for early child development implementation: multicountry mixed methods evaluation. *BMJ Open* 2020;**10**:e032134. doi:10.1136/bmjopen-2019-032134

22 Gladstone M, Phuka J, Mirdamadi S, *et al.* The care, stimulation and nutrition of children from 0-2 in Malawi—Perspectives from caregivers; ‘Who’s holding the baby?’ *PLOS ONE* 2018;**13**:e0199757. doi:10.1371/journal.pone.0199757

23 Kumar V, Kumar A, Ghosh AK, *et al.* Enculturating science: Community-centric design of behavior change interactions for accelerating health impact. *Semin Perinatol* 2015;**39**:393–415. doi:10.1053/j.semperi.2015.06.010

24 Engle PL, Fernald LC, Alderman H, *et al.* Strategies for reducing inequalities and improving developmental outcomes for young children in low-income and middle-income countries. *The Lancet* 2011;**378**:1339–53. doi:10.1016/S0140-6736(11)60889-1

25 McCoy DC, Seiden J, Cuartas J, *et al.* Estimates of a multidimensional index of nurturing care in the next 1000 days of life for children in low-income and middle-income countries: a modelling study. *Lancet Child Adolesc Health* 2022;**6**:324–34. doi:10.1016/S2352-4642(22)00076-1

For peer review only - http://bmjopen.bmj.com/site/about/guidelines.xhtml

607 26 Seiden J, Pisani L, Cuartas J, *et al.* Different is not deficient: respecting diversity in early childhood development – Authors' reply. *Lancet Child Adolesc Health* 2022;**6**:e27. doi:10.1016/S2352-4642(22)00307-8

610 27 Black MM, Richter LM. Different is not deficient: respecting diversity in early childhood development. *Lancet Child Adolesc Health* 2022;**6**:e26. doi:10.1016/S2352-4642(22)00276-0

613 28 Lingam R, Gupta P, Zafar S, *et al.* Understanding care and feeding practices: building blocks for a sustainable intervention in India and Pakistan. *Ann N Y Acad Sci* 2014;**1308**:204–17. doi:10.1111/nyas.12326

616 29 Hollowell J, Belem M, Swigart T, *et al.* Age-related patterns of early childhood development practices amongst rural families in Burkina Faso: findings from a nationwide survey of mothers of children aged 0-3 years. *Glob Health Action* 2020;**13**:1772560. doi:10.1080/16549716.2020.1772560

620 30 Jeong J, Pitchik HO, Yousafzai AK. Stimulation Interventions and Parenting in Low- and Middle-Income Countries: A Meta-analysis. *Pediatrics* 2018;**141**:e20173510. doi:10.1542/peds.2017-3510

623 31 Nair S, Chandramohan S, Sundaravathanam N, *et al.* Father Involvement in Early Childhood Care: Insights From a MEL System in a Behavior Change Intervention Among Rural Indian Parents. *Front Public Health* 2020;**8**.https://www.frontiersin.org/articles/10.3389/fpubh.2020.00516 (accessed 11 Aug 2022).

628 32 Rothstein JD, Buckland AJ, Gagnier K, *et al.* Assessing the play and learning environments of children under two years in peri-urban Lima, Peru: a formative research study. *BMC Public Health* 2021;**21**:108. doi:10.1186/s12889-020-10119-3

631 33 Zhang D, Jin L, Liang D, *et al.* Assessing Feasibility of an Early Childhood Intervention Using Mobile Phones Among Low-Income Mothers of Newborns: Qualitative Interview Study. *JMIR Form Res* 2020;**4**:e17179. doi:10.2196/17179

634 34 Strachan D, Kabwijamu L, Okuga M, *et al.* Shifting the discourse from survive to thrive: a qualitative exploration of beliefs, actions and priorities for early childhood development in Uganda. *J Br Acad* 2020;**8s2**:41–70. doi:10.5871/jba/008s2.041

637 35 Singla DR, Kumbakumba E, Aboud FE. Effects of a parenting intervention to address maternal psychological wellbeing and child development and growth in rural Uganda: a community-based, cluster-randomised trial. *Lancet Glob Health* 2015;**3**:e458–69. doi:10.1016/S2214-109X(15)00099-6

641 36 Mebrahtu H, Simms V, Mupambireyi Z, *et al.* Effects of parenting classes and economic strengthening for caregivers on the cognition of HIV-exposed infants: a pragmatic cluster randomised controlled trial in rural Zimbabwe. *BMJ Glob Health* 2019;**4**:e001651. doi:10.1136/bmjgh-2019-001651

For peer review only - http://bmjopen.bmj.com/site/about/guidelines.xhtml

37 Barnhart DA, Farrar J, Murray SM, *et al.* Lay-worker Delivered Home Visiting Promotes Early Childhood Development and Reduces Violence in Rwanda: A Randomized Pilot. *J Child Fam Stud* 2020;**29**:1804–17. doi:10.1007/s10826-020-01709-1

38 Ministry of Health Rwanda, UNICEF Rwanda. Knowledge, Attitudes and Practices Assessment on Early Nurturing of Children Report. 2014. https://silo.tips/queue/knowledge-attitudes-practices?&queue_id=-1&v=1680203477&u=NzMuMTg2LjEzOC4yMjg=

39 The Manoff Group. Trials of Improved Practices (TIPS): Giving Participants a Voice in Program Design. 2005.https://www.behaviourchange.net/document/52-trials-of-improved-practices-tips-giving-participants-a-voice-in-program-design

40 Weber AM, Diop Y, Gillespie D, *et al.* Africa is not a museum: the ethics of encouraging new parenting practices in rural communities in low-income and middle-income countries. *BMJ Glob Health* 2021;**6**:e006218. doi:10.1136/bmjgh-2021-006218

41 Feldman HM. How Young Children Learn Language and Speech. *Pediatr Rev* 2019;**40**:398–411. doi:10.1542/pir.2017-0325

42 Prochner L, Kabiru M. ECD in Africa: A historical perspective. 2008.https://documents1.worldbank.org/curated/en/135791468211777082/pdf/427000PUB0Afri1sclosed0Feb025020081.pdf

43 Murray L, Cooper P, Arteche A, *et al.* Randomized controlled trial of the effect of a home visiting intervention on infant cognitive development in peri-urban South Africa. *Dev Med Child Neurol* 2015;**58**:270–6. doi:10.1111/dmcn.12873

44 London School of Hygiene and Tropical Medicine. SUNRISE Cluster RCT: Scaling Up Nurturing Care, a Radio Intervention to Stimulate Early Child Development in Burkina Faso. clinicaltrials.gov 2022. https://clinicaltrials.gov/ct2/show/NCT05335395 (accessed 9 Jan 2023).

45 Nsamenang, A. Bame. In a globalised world, local culture must be the anchor of identity. *Early Child Matters* 2014.

46 Serpell R. Africentric engagement with child development theory and education: the legacy of Bame Nsamenang. *Int J Behav Dev* 2019;**2019**:3–4.

47 Modica V Sarah;Ajmera, Maya;Dunning. Culturally Adapted Models of Early Childhood Education. *YC Young Child* 2010;**65**.

48 Bentley ME, Johnson SL, Wasser H, *et al.* Formative research methods for designing culturally appropriate, integrated child nutrition and development interventions: an overview. *Ann N Y Acad Sci* 2014;**1308**:54–67. doi:10.1111/nyas.12290

49 Chambers DA, Glasgow RE, Stange KC. The dynamic sustainability framework: addressing the paradox of sustainment amid ongoing change. *Implement Sci IS* 2013;**8**:117. doi:10.1186/1748-5908-8-117

50 Lanyasunya AR, Lesolayia MS. *The El-Barta Child and Family Project--Community Based Early Child Care and Development Programme: An Integrated Approach. Working Papers in*

For peer review only - http://bmjopen.bmj.com/site/about/guidelines.xhtml

Early Childhood Development. Bernard van Leer Foundation, P 2001. https://eric.ed.gov/?id=ED460778 (accessed 7 Sep 2022).

51 Institut National de la Statistique et de la Démographie. Burkina Faso Enquête Démographique et de Santé 2021 (French). 2022. https://dhsprogram.com/publications/publication-PR139-Preliminary-Reports-Key-Indicators-Reports.cfm (accessed 4 Aug 2023).

52 Bergen N, Labonté R. “Everything Is Perfect, and We Have No Problems”: Detecting and Limiting Social Desirability Bias in Qualitative Research. *Qual Health Res* 2020;**30**:783–92. doi:10.1177/1049732319889354

53 LaMonica HM, Crouse JJ, Song YJC, *et al.* Developing a Parenting App to Support Young Children’s Socioemotional and Cognitive Development in Culturally Diverse Low- and Middle-Income Countries: Protocol for a Co-design Study. *JMIR Res Protoc* 2022;**11**:e39225. doi:10.2196/39225

For peer review only

Guide for In-depth interviews with Families on Care-giving

Welcome. We would like to understand more about how young children are cared for in your community to help us design a radio campaign that focuses on child development.

We would like to learn from you about current care giving practices, what you believe makes your child healthy and what your hopes are for your children.

[Reminder to interviewer: Questions should relate to the focus child less than 3 years of age]

Theme 1: Socio-demographic and interview information

- 1.1 ID:
- 1.2 Interview date:
- 1.3 Interview start time:
- *1.4* Interview end time:
- 1.5 Age:
- *1.6* Marital status:
- 1.7 Ethnicity/religion:
- 1.8 Last completed level of education:
- 1.9 Main occupation:
- 1.10 Total number of children:
- 1.11 Sex of baby:
- 1.12 Age of baby:
- 1.13 Interviewer code:
- 1.14 Tape recording number:

Theme 2: Typical day

1. Who has the main responsibility of caring for your child? What responsibilities do they have?

2. Who else is the child with during a typical day? How often and how long for? What do they do with the child?
   a. **Probe:** role of grandmother, father, siblings, neighbours

3. What are the key events in your child's day? [e.g. bathing, massage/ lotion, feeding].

4. Would you like to change the amount of time you spend with your child (more? Less?)
   a. **Probe:** *If more*: What are the reasons you spend less time than you would like with your child? What would you like to do if you had more time?

Theme 3: Learning and playing

1. From what age do you think your child begins to learn new things?
   a. When do they start to learn and understand about the world around them?
   b. When do they start to understand words?

2. I would like to know a little bit about how your child learns new things?
   a. How does your child learn new behaviours or skills?
   b. How does your child learn what is good or wrong (moral values)?
   c. Hoes does your child learn new words and sounds?
   d. At what age will they learn most? Learn the quickest?

3. What are you excited about seeing them learn or do in the next few years?

For peer review only

4. Tell me about the times you play with your child.
    a. When?
    b. What or how do you play?

5. What are your child's favourite things to play or to play with?

6. Does anyone else play with your child?
    a. Who?
    b. When?
    c. What or how do they play?

7. Does your child play on their own?
    a. When?
    b. What or how do they play?

8. What do you think about using household things such as bowls, bottles and spoons as play things?

9. Do you think your child learns anything while they play?
    a. If yes, what do you think your child learns when she/he plays?

10. Tell me about the times you talk to your child?
    a. What type of things do you talk to them about?
    b. Are they able to communicate back?

11. Do you tell your child stories/songs/rhymes?
    a. Why/why not?
    b. Can you share some of your favourite stories/songs/rhymes with me?

12. Do you look at books with your child?
    a. If you wanted to show your child some pictures of people, things or places what could you use?

Theme 4: Responsive caregiving

1. How do you make your child smile/laugh?

2. How do you show them that you love them?

3. How does your child let you know what they want or need?
    a. What do you do when they show you these things?

4. What sort of things make you unhappy with him/her?
    a. Can you share an example of the last time you were unhappy with your child?
    b. What did you do?

5. If your child does something good, how do you encourage them to do it again?

For peer review only

6. If your child does something that is unsafe, like get too close to the fire, how do you help them understand this is dangerous?

7. How is your child disciplined?
    a. Who disciplines them?
    b. Does everyone in the family agree about how to disciplined the child?

Theme 5: Aspirations and challenges

1. We all have hopes for our children's futures. What do you hope for your child as they grow older? What does a successful life look like for your child?

2. What can you do with your child to help them be that way? To meet those goals?

3. What kind of parent/caregiver do you want to be for your child?
    a. What problems to you face trying to be this type of parent?

4. Do you have any worries/concerns about your child's future?

5. Do you face any challenges when caring for your child? If yes, can you describe those challenges?

Theme 6: Additional notes for interviewer

1. Interviewer note here:
    a. Where interview took place
    b. Any interruptions
    c. Overall mood, openness, and engagement of participant

For peer review only

<u>Draft Guide for focus group discussions with Families on Care-giving -FGD 1</u>

Welcome. We would like to understand more about how young children are cared for in your community to help us design a radio campaign that focuses on child development.

We would like to discuss how children are cared for in this community

Theme 1: Socio-demographic and interview information

1.1 FGD ID:
1.2 FGD date (DD/MM/YYYY):
1.3 Village:
1.4 FGD start time (AM/PM):
1.5 FGD end time (AM/PM):
1.6 Interviewer code:
1.7 Note taker code
1.8 Tape recording number:

Respondent number	Age	Marital Status	Education	Ethnicity/ religion	Number of children	Age of youngest child

Theme 2: General child care

1. In general, who is responsible for caring for children the age of your child in this community? [Probes: Feeding, bathing, keeping them safe]

2. What role do fathers play in the care of children the age of your child? Do you think fathers would like to spend more or less time with their children?

3. What would the community say about a father who spent a lot of time with their young child? Would they see this as a good thing? A bad thing?

Theme 3: Playing

1. When do children start to learn?

2. What does it mean when a child is called smart? How does a child become smart?

3. At what age do children begin to play?

4. Is play important for a child? Why? [Probe: What do you think your child learns when she/he plays?]

5. What do you think would happen if people in this community started praising their child more for being 'good' and punished them less for being 'bad'.

6. How would you describe a 'difficult' (troublesome) child? How does a 'difficult' child behave? What, generally, are the behaviors of a 'difficult' child? How would you say a child becomes 'difficult'?

For peer review only

Theme 4: Radio clips

We would now like you to listen to three radio clips and we are interested to know what you think of these clips, whether you would want to do the activities described in them with your child or not. There is no right or wrong answer, we are only interested in your thoughts, so please be honest.

Follow up questions clip 1:

1. What do you think about the information shared in this clip?
2. Did you hear information that was new to you in this clip? What?
3. How does what you heard compare to what you currently do with your children?

Follow-up questions clips 2-4*:*

We just heard information about ***talking*** *to your child,* ***playing*** *with your child and* ***praising*** *your child.*

1. What do you think about the information shared in this clip?
2. Did you hear information that was new to you in this clip? What?
3. How does what you heard compare to what you currently do with your children?
4. Would anyone in your family or the community think any of these suggestions was bad or negative for your child if you did them? Why or why not?

We heard descriptions of three activities that you can do with your child to help them grow and develop. The three activities were:

1. *Talk to your child throughout the entire day. For example, you can talk to your baby during bathing, dressing, breastfeeding, diaper changes, chores (such as cooking, sweeping, and washing clothes), when sitting with the family.*
2. *Play with your child at least 15 minutes a day. Your child can play with things you have around the house - like bowls, spoons or cups - or toys you buy.*
3. *Encourage and congratulate your child. Encourage, applaud or congratulate your child when they do something that you like or learn a new skill. Hug and cuddle them a lot.*

We would like you to try these activities at least once a day. However, if you cannot do the activity every day – or at all – that is ok! This is not a test. We will meet in one week from today to discuss with you how the week went. We want to learn more about your experiences, even if you cannot do the activity every day or at all.

Next week we are going to meet here again and discuss how it went practising these suggestions with your baby/child.

Do you have any questions?

For peer review only

For peer review only

Draft Guide for focus group discussions with Families on Care-giving -FGD 2

Welcome. We are going to discuss you experiences trying the behaviours we heard about last week. The behaviours we asked you to try can be difficult to do during your busy week – or maybe your child did not like them. We want to hear about any of your experiences, even if you were not able to practice with your child.

Theme 1: Socio-demographic and interview information

a. FGD ID:
b. FGD date (DD/MM/YYYY):
c. Village:
d. FGD start time (AM/PM):
e. FGD end time (AM/PM):
f. Interviewer code:
g. Note taker code
h. Tape recording number:

Respondent number	Ag e	Marital Status	Education	Ethnicity/ religion	Number of children	Age of youngest child

1. We asked you to try some behaviours at home, how would you describe what you did differently?

2. Were there any behaviours you were not able to practise with your child or that were difficult to do?
   a. If yes, which behaviours?
   b. Why or why not? [probe to identify key challenges]

3. How did your child react to the behaviours?

4. Do you think doing these activities will help your child in any way? Why/why not? How?

5. How did you feel while talking with, playing with or praising your child?
   a. [**Probe**: Enjoyable? Fun? Neutral? Difficult? Annoying? Strange?]

6. How did your family members react?
   a. [**Probe**: other children? Spouse, co-wives, grandparents?].

7. Do you feel like you could incorporate the behaviours into your routine every day?
   a. If yes, when could you practise these behaviours with your child?
   b. Which ones would be easiest? Which was the hardest? Why?

8. Thinking about other families in your community, what is the main difficulty they will face in behaving in the ways we asked you to try?

9. Are there any behaviours they will not want to do?

For peer review only

Supplemental material

'Children awaken by playing': A qualitative exploration of caregivers' perceptions of young children's learning and early childhood development in rural Burkina Faso

Audio clips played for focus group discussion participants

→ The following text was audio recorded in French, Bissa and Dioula and played for participants in Focus Group Discussions. Participants were then asked for their reactions to the information shared in the recordings.

Clip 1: The basics and benefits of early childhood development

Children are curious from the start of their lives. They want to learn and understand the world. During the first five years of life, a child's brain grows faster than at any other time. So anything you say and do can help build a child's brain. Children's early experiences determine their brain development and give them the foundation for success in life. Talking and playing with the child from birth will make him active and intelligent.

Clip 2: Talk

Your children get it before they start talking. When they babble and twitter, they are looking to communicate or 'talk' in their own way with you. Talk to them often and imitate their sounds and expressions to see how they react - each of you can speak in turn!

Your baby loves the sound of your voice. Talk to your baby about everything you do during the day. Describe what you hear, see and smell around you. Point to objects and people and name them. This will facilitate their learning. You can talk to your baby during the bath, while you are dressing her, during meals, when you breastfeed her. You can also do this during household chores (such as cooking, cleaning, and laundry), or when you're sitting with your family and before bedtime. Tell your family to talk to the baby often, too. The more the baby is exposed to language (even before he can speak), the more words he will learn!

Clip 3: Play

It is through play that children learn about the world around them. Give your child your full attention for at least 15 minutes a day. Encourage him to look at your face or at the colored objects around you such as a picture, a plate or a mug.

When your baby gets older, help him discover the world around him by giving him items to hold in his hand to play. Look for safe objects or toys that he can hold in his hands and that he can shake or roll (such as a lime, orange, or any other round object in the house). You can also put beans in a small container with a lid that the baby can use and shake like a rattle. Have fun with him by hiding your face

behind your hands and making him laugh. This will help her understand that things don't go away even if they are hidden.

Take part in your child's play and exploration whenever you can. That way when he gets stuck or gets frustrated, you can help him kindly.

Clip 4: Encouragement and praise

Encouraging your child, at any age, shows him that you are happy with what he has done and it will make him want to do it again. Your child wants to know that you are happy with what he has done and the effort he has put in. Therefore, he will learn faster when you encourage him to try new things. It's always better to encourage your child to do the things you want them to do rather than constantly telling them not to do the things you don't want them to do! So encourage and praise him rather than saying negative words like “bad” or “don't do that.

“Instead, say “well done” and “well done!”, clap or smile when he does something that you like or when he is learning something new. Even babies who have not yet learned to speak are encouraged with positive words. So encourage and praise your baby when he tries to do new things (if he picks up objects or holds them in his hand, if he claps with his hands or tries to stand unaided). Use hugs, kisses and loving words with your child every day. Your child will then be brought in more often to do the activities you want him to do.

Table 1. Participant socio-demographics

Research Activity	Participant group	Total number of participants	Participant age range	Range of number of children**	Age ranges of children**			Child gender***	
					0-11 mo	*12-23 mo*	*24+ mo*	*Girl*	*Boy*
In-depth interviews	Mothers	16	20-40	1-7	6	8	2	9	7
	Fathers	16	23-59	1-15	8	7	1	6	10
*Focus Group Discussions**	Mothers	28	18-36	1-6	13	8	0		
	Fathers	26	24-58	1-10	17	9	0		
	Grandmothers	27	40-68	1-10	20	7	0		

* 5-7 participants per FGD
** For Grandmothers, refers to number/ ages of grandchildren
***Child gender only collected for IDIs

For peer review only

COREQ (COnsolidated criteria for REporting Qualitative research) Checklist

A checklist of items that should be included in reports of qualitative research. You must report the page number in your manuscript where you consider each of the items listed in this checklist. If you have not included this information, either revise your manuscript accordingly before submitting or note N/A.

Topic	Item No.	Guide Questions/Description	Reported on Page No.
**Domain 1: Research team and reflexivity**			
Personal characteristics			
Interviewer/facilitator	1	Which author/s conducted the interview or focus group?	
Credentials	2	What were the researcher's credentials? E.g. PhD, MD	
Occupation	3	What was their occupation at the time of the study?	
Gender	4	Was the researcher male or female?	
Experience and training	5	What experience or training did the researcher have?	
Relationship with participants			
Relationship established	6	Was a relationship established prior to study commencement?	
Participant knowledge of the interviewer	7	What did the participants know about the researcher? e.g. personal goals, reasons for doing the research	
Interviewer characteristics	8	What characteristics were reported about the inter viewer/facilitator? e.g. Bias, assumptions, reasons and interests in the research topic	
**Domain 2: Study design**			
Theoretical framework			
Methodological orientation and Theory	9	What methodological orientation was stated to underpin the study? e.g. grounded theory, discourse analysis, ethnography, phenomenology, content analysis	
Participant selection			
Sampling	10	How were participants selected? e.g. purposive, convenience, consecutive, snowball	
Method of approach	11	How were participants approached? e.g. face-to-face, telephone, mail, email	
Sample size	12	How many participants were in the study?	
Non-participation	13	How many people refused to participate or dropped out? Reasons?	
Setting			
Setting of data collection	14	Where was the data collected? e.g. home, clinic, workplace	
Presence of non-participants	15	Was anyone else present besides the participants and researchers?	
Description of sample	16	What are the important characteristics of the sample? e.g. demographic data, date	
Data collection			
Interview guide	17	Were questions, prompts, guides provided by the authors? Was it pilot tested?	
Repeat interviews	18	Were repeat inter views carried out? If yes, how many?	
Audio/visual recording	19	Did the research use audio or visual recording to collect the data?	
Field notes	20	Were field notes made during and/or after the inter view or focus group?	
Duration	21	What was the duration of the inter views or focus group?	
Data saturation	22	Was data saturation discussed?	
Transcripts returned	23	Were transcripts returned to participants for comment and/or	

For peer review only

Topic	Item No.	Guide Questions/Description	Reported on Page No.
		correction?	
**Domain 3: analysis and findings**			
Data analysis			
Number of data coders	24	How many data coders coded the data?	
Description of the coding tree	25	Did authors provide a description of the coding tree?	
Derivation of themes	26	Were themes identified in advance or derived from the data?	
Software	27	What software, if applicable, was used to manage the data?	
Participant checking	28	Did participants provide feedback on the findings?	
Reporting			
Quotations presented	29	Were participant quotations presented to illustrate the themes/findings? Was each quotation identified? e.g. participant number	
Data and findings consistent	30	Was there consistency between the data presented and the findings?	
Clarity of major themes	31	Were major themes clearly presented in the findings?	
Clarity of minor themes	32	Is there a description of diverse cases or discussion of minor themes?	

Developed from: Tong A, Sainsbury P, Craig J. Consolidated criteria for reporting qualitative research (COREQ): a 32-item checklist for interviews and focus groups. *International Journal for Quality in Health Care*. 2007. Volume 19, Number 6: pp. 349 – 357

Once you have completed this checklist, please save a copy and upload it as part of your submission. DO NOT include this checklist as part of the main manuscript document. It must be uploaded as a separate file.

For peer review only
